# A checklist of rheophytes of Cameroon

**DOI:** 10.3897/phytokeys.121.29924

**Published:** 2019-05-09

**Authors:** Felix Kuetegue, Bonaventure Sonké, Gabriel K. Ameka

**Affiliations:** 1 Plant Systematic and Ecology Laboratory, Higher Teachers´ Training College, University of Yaoundé I, P.O. Box 047, Yaoundé, Cameroon University of Yaoundé I Yaounde Cameroon; 2 International Joint Laboratory DYCOFAC, IRD-UYI-IRGM, BP1857, Yaoundé, Cameroon International Joint Laboratory DYCOFAC Yoaundé Cameroon; 3 Department of Plant and Environmental Biology, University of Ghana, P. O. Box LG 55, Legon, Accra, Ghana University of Ghana Accra Ghana

**Keywords:** Cameroon, conservation, distribution, inventory, Rheophyte diversity

## Abstract

Rivers in Cameroon were surveyed to collect and document rheophytic plants. Rheophytes are the dominant aquatic macrophytes in tropical river systems, where they are adapted to extreme environments of rushing water (e.g., river rapids, waterfalls and flash floods). Rheophytic plants are useful indicators of river health. However, their habitats are threatened by human activities such as agriculture, plantation development, alluvial mining and dam construction, particularly in tropical countries. In this survey we documented 66 rheophytic species in 29 genera and 16 families. Two ferns, 8 monocotyledons and 56 dicotyledons were listed. Apart from the Podostemaceae family in which all species are rheophytic, the other 15 families have few species which are rheophytic. Five of these families have up to four species and the remaining 10 have only one member as a rheophytic species. The conservation status of each species is assessed and discussed. This work urges botanists, conservationists, and policy makers to do more to protect the habitats of rheophytes and put in place strategies and action plans for the conservation of this important biological group.

## Introduction

Rheophyte, a term coined by van Steenis in 1932 (van Steenis 1978), is used to describe an aquatic plant which is in nature restricted to swift-running rivers and streams and grows up to flood level, but not beyond the reach of regularly occurring flash floods (van Steenis 1978, 1981). Rheophytes occur worldwide but are found particularly in evergreen rain forests, where they are the dominant aquatic macrophytes in tropical river systems (van Steenis 1978, Quiroz et al. 1997, Ameka 2000, Hoyos-Gomez and Bernal 2018). Members of this biological group of plants are not necessarily taxonomically related, but they show a common adaptation to a restricted ecological habitat or environmental factors (van Steenis 1981, Ameka 2000, Ameka et al. 2002, Hoyos-Gomez and Bernal 2018). Rheophytes are adapted to extreme environments of rushing water by having lanceolate leaves, slender and flexible but tough stems, and strong usually fibrous, root systems (Hoyos-Gomez and Bernal 2018). These plants are generally perennial herbs or shrubs, sometimes small to medium trees, while few grow into tall trees. Two categories of rheophytes are recognized: (i) obligate and, (ii) facultative rheophytes (e.g., Ameka et al. (2002)). Obligate rheophytes are confined to waterfalls, streams and river-beds and banks, and below the flood level. Facultative types are found not only in river-beds but also occur in wet places where they are not subjected to fast-flowing water. In this work, rheophytic plants or rheophytes refer to obligate rheophytes.

Twenty-one rheophytic species, excluding the Podostemaceae, were recognized in tropical Africa, in a worldwide census of rheophytes by van Steenis (1981). Earlier in an assessment of rheophytic plants in South Africa, van Steenis (1978) recognized 7 species, again excluding the Podostemaceae. According to Ameka et al. (2002), in a survey of rivers, for rheophytes, in southern Ghana, from 1994 to 2000, 15 species including four Podostemaceae were recorded. Surprisingly, woody rheophytes were not encountered in the survey by Ameka et al. (2002) although van Steenis (1981) had earlier indicated that half of all rheophytes worldwide are woody. In their work on rheophytes of southern Ghana, Ameka et al. (2002) also reviewed rheophytes of Africa but relied only on records from the literature. Their review revealed that ca. 114 species including 73 Podostemaceae species were documented as rheophytes in Africa. Regarding distribution by country, Cameroon was reported to have 53 rheophytes including 33 Podostemaceae; South Africa 7 rheophytes and 3 Podostemaceae; and Nigeria 19 rheophytes including 4 Podostemaceae (Ameka et al. 2002). The known number of rheophytic plants recorded for Africa (including Cameroon, Ghana, Nigeria and South Africa) probably underestimates the actual number and reflects the degree of paucity of information, and lack of systematic collection effort of Podostemaceae and other rheophytic plants in African countries.

A survey to document and study the rheophytes of Cameroon is important for a number of reasons: (i) rheophytes are poorly known in tropical Africa, including Cameroon, compared to South East Asia and South America, according to van Steenis (1981), (ii) they are the dominant aquatic macrophytes in rivers; and are useful biological indicators of river health, and (iii) the diversity of rheophytes is threatened and some species are in danger of disappearing by the increased land-use practices adjoining the rivers, particularly for agriculture, plantation development, and illegal logging; and in the river courses for alluvial mining (e.g., gold and diamond), and also damming of rivers for hydropower in tropical countries. The construction of dams causes destruction of the habitats of rheophytes, particularly the Podostemaceae.

The survey to enumerate and document the rheophytes of Cameroon was conducted from 2010 to 2014; and the rheophytic species encountered are reported here. It is hoped that this work will stimulate further research on rheophytes across the rest of tropical Africa. We draw attention to the urgent need to stop the destruction of habitats of rheophytes and rather map out strategies and action plans for the conservation of this important biological group.

## Materials and methods

### The study site

**Figure 1.**
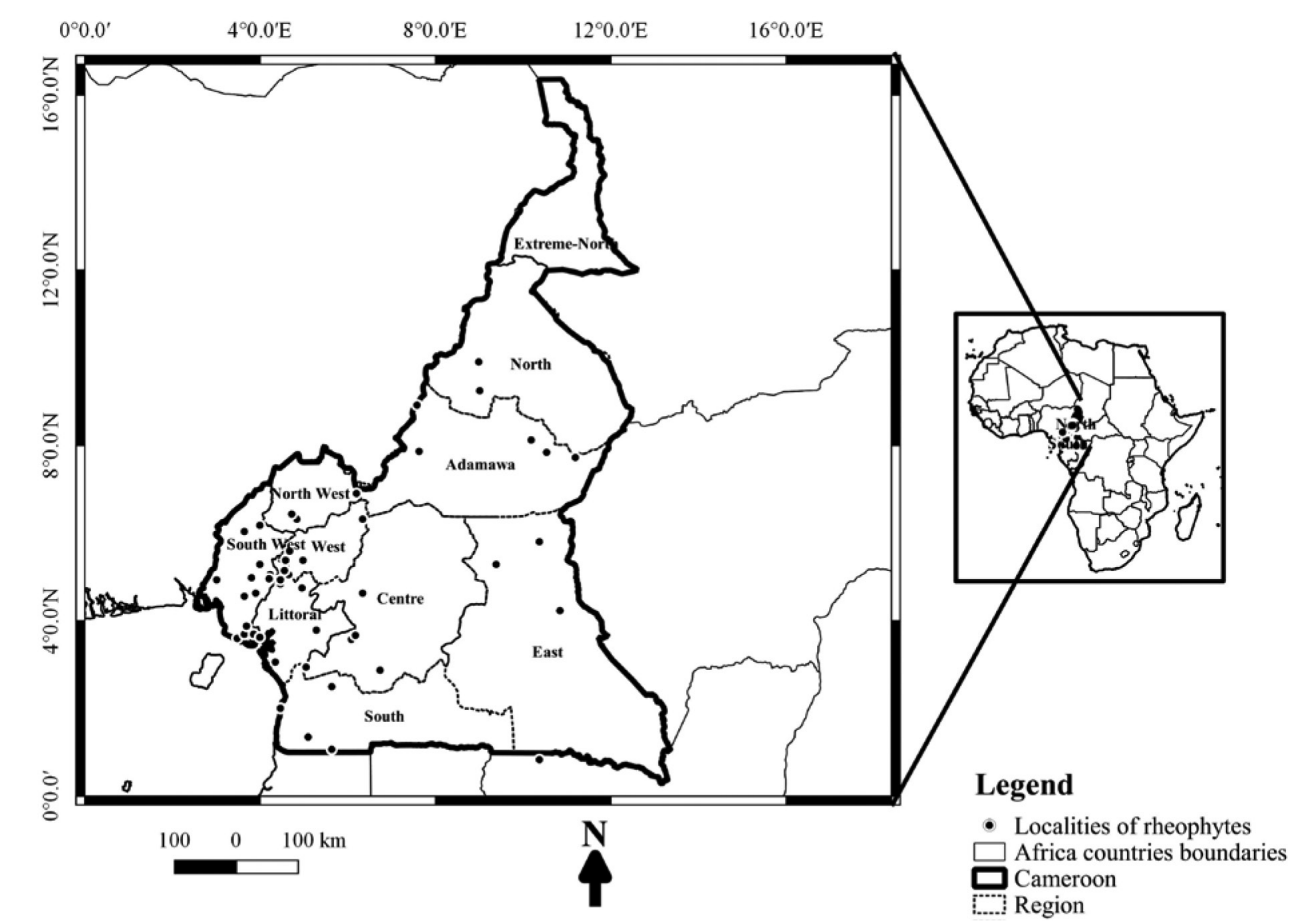
Map of Cameroon showing collecting localities of rheophytes.

A survey of rheophytes was carried out in Cameroon, situated between 2°–13°N and 9°–16°E (Fig. 1). Cameroon is generally divided into three main climatic zones: Equatorial climate zone, (2°–6°N), characterized by an annual average precipitation of 2000 mm, and an average temperature of about 25 °C; the Sudanese climate zone, (6°–10°N), characterized by 5–6 months of dry season with an average temperature of about 22 °C, and 1000 mm of precipitation; and the Sudano-Sahalian climate zone, (10°N–13°N), characterized by 7 months of dry season and 400–900 mm of precipitation (Olivry 1986, Munang et al. 2008). From north to south Cameroon, the vegetation ranges from steppe zone, savannah zone, and to forest zone.

The central and western parts of Cameroon are dominated by high mountains and plateaus (Segalen 1967, Suchel 1972, Ndoh et al. 2016). The high western range has peaks which vary in elevation e.g., Mt Etinde (1474 m), Mt Mwoanenguaba (2396 m), Mt Kupe (2050 m), Mt Bamboutos (2740 m) with the highest elevation at Mt Cameroon (4095 m) (Letouzey 1985, Cheek et al. 2004, Sainge 2017). The Adamawa or Central High plateau reaches up to 1500 m (Suchel 1972). Both the western range and the Central High plateau are the result of volcanic and tectonic activities giving rise to faults, volcanic cones and volcanic lakes. These two sectors constitute the main watersheds of Cameroon’s drainage systems. The southern section of the country is dominated by a plateau (500 to 900 m) which gently slopes to the east (Congo basin) but falls by steps to the Atlantic coast (Suchel 1972). While the far north is dominated by the lake Chad Basin (Suchel 1972, Olivry 1986), its southern fringe is the River Benue basin, both of which present a monotonous relief. The narrow coastal zone is marked by unstable swamps, especially from the Wouri-Moungo basins to the Ndian-Akpa-Yafe basins (Suchel 1972, Olivry 1986).

### Survey

In documenting the rheophytes of Cameroon, several rivers (Fig. 1) were visited during the dry season (November-February and July–August in the southern part of the country; and October–April in the northern part of the country). In the dry period, water levels recede in rivers and the majority of plants are in their reproductive phase. Sections of the rivers with rocky substrate were intensively sampled. The rheophytic status of some species were in doubt, particularly those on river banks and edge of rivers, such species and their localities were visited also during the wet season (March –June and July–October in the southern part of the country; May–September in the northern part of the country), when the water flow and level were high. This enabled us to determine whether a particular species is able to withstand spate, and is therefore rheophytic. Notes were taken on the habitat conditions, and characteristic rheophytic features of the species encountered, particularly the leaf shape and form, stem characteristics, and rooting system. Voucher specimens of each taxon collected were deposited in the Cameroon National Herbarium in Yaoundé (YA), [YA, acronym, Thiers (2017)]. Voucher specimens were not collected for every rheophyte species (particularly the Podostemaceae) encountered in the field. This is because such species were common and voucher specimens already exist for them. In cases where voucher specimens were not collected, notes were taken to indicate presence of the rheophytic species at the locality.

Voucher specimens in YA were also consulted for rheophytes already collected from Cameroon. The Flora of West Tropical Africa (Keay 1954, 1958, Hepper 1963, 1968, 1972) and other published works on rheophytes (e.g., van Steenis 1978, van Steenis 1981, Ameka et al. 1996, Ameka, et al. 2002), and from the study area (e.g., Hooper (1972), Duncan (1986), Cusset (1987), Cable and Cheek (1998), De Block (1998), Pollard and Paton (2001), Cheek et al. (2004), Brooks et al. (2011), Ghogue (2011), Onana and Cheek (2011), Cheek et al. (2017a), and Cheek et al. (in press)), were also referred to, and used to confirm the identification of rheophyte species collected, and in the compilation of a rheophytic checklist for Cameroon. In addition, the following websites were also consulted: http://www.worldchecklistofplants.org; http://www.ipni.org; http://www.plantlist.org; http://www.iucnredlist.org.

Distribution maps of rheophytes of Cameroon were done using georeferenced specimen data derived from specimen labels or available literature, and our own field surveys. The conservation status of each species was assessed by calculating the extent of occurrence (EOO) and the area of occupancy (AOO) in Cameroon using GeoCAT (Geospatial Conservation Assessment tool; Bachman et al. 2011) and applying The IUCN Red List Categories and Criteria, version 10.1 (IUCN 2013, IUCN Standards and Petitions Subcommittee 2017). The AOO was calculated based on a user defined grid cell of 2 km. The number of ‘locations’ (as defined by IUCN 2017) was calculated with regard to each particular threat, such that a single ‘location’ may encompass more than one adjacent subpopulation. The term subpopulation is used according to IUCN (2017). The Red Data Book of the Flowering Plants of Cameroon: IUCN Global Assessments (Onana and Cheek 2011), Ghogue (2011), and the online IUCN Red List (http://www.iucnredlist.org) were consulted while determining the conservation status of the species in this study. Our conservation assessments are yet to be submitted to IUCN and as such these assessments should be treated as “preliminary conservation assessments”.

## Results

The list of rheophytes identified during the study is presented as a checklist organized by families, and each entry consists of the following:

- Species name, authority and place of publication
- Synonym(s) where applicable
- Type, followed by Basionym where applicable
- Description
- Specimens examined
- Habitat
- Distribution
- Conservation status in Cameroon

### Checklist of rheophytes from Cameroon

The checklist of rheophytes of Cameroon contains 16 families and 66 species. The rheophytic species listed may be placed in two categories: in the first category are families in which few species are rheophytic and in the second category are families in which all species are rheophytic. The former category has 15 families, 17 genera, and 23 species, while the latter category contains only the Podostemaceae family with 12 genera and 43 species.

### Families in which few species are rheophytic

#### Pteridophytes (ferns)

##### Lomariopsidaceae

###### Bolbitis fluviatilis

Taxon classificationPlantaePolypodialesLomariopsidaceae

(Hook.) Ching, Index Filic. Suppl. Tert. 48 (1934)

Acrostichum fluviatile Hook., Sp. Fil. 5: 274 (1864)
Acrostichum phanerodictyon Baker, Bol. Soc. Brot. 4: 156, t. 2 (1886)
Leptochilus fluviatilis (Hook.) C.Chr., Index Filic. 10, 385 (1905)

####### Type.

Equatorial Guinea, Fernando Po (Bioko), *G. Mann 442* (K, K000435773).

####### Description.

Herbaceous, rhizome creeping, with opaque, castaneous scales; fixed to rocks by roots; sterile fronds lanceolate, up to 85 cm long; fertile fronds up to 90 cm long with sporangia on lower surface.

####### Specimens examined.

15 km southeast of Zingui, 14 Mar 1968, *R. Letouzey 9031* (YA); near Ababendoman, 65 km southeast of Ebolowa, 00 Jan 1970, *R. Letouzey 9958* (YA); Muanenguba Mts. northeast of Nkongsamba, 4°58'N, 9°53'E, 11 Dec 1971, *A. J. M. Leeuwenberg 8848* (YA).

####### Habitat.

Rocky riverbeds and streams, and rocky borders of streams and rivers; in evergreen rainforest.

####### Distribution.

**Figures 2–10.**
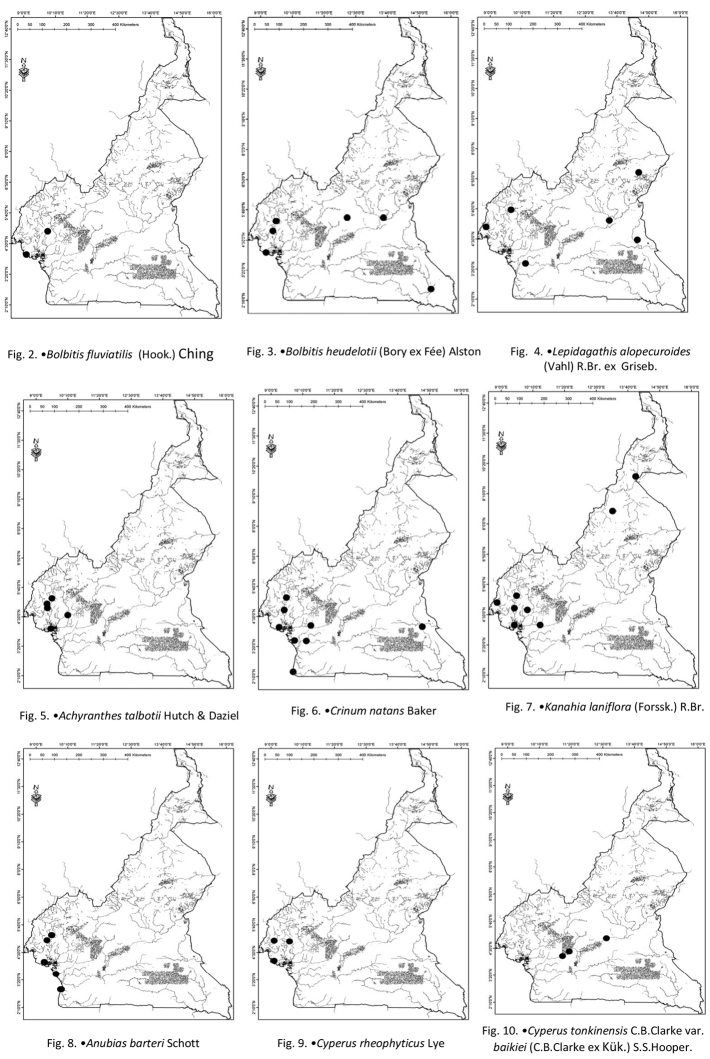
**2***Bolbitisfluviatilis* (Hook.) Ching **3***Bolbitisheudelotii* (Bory ex Fée) Alston **4***Lepidagathisalopecuroides* R.Br. ex Griseb. **5***Achyranthestalbotii* Hutch & Daziel **6***Crinumnatans* Baker **7***Kanahialaniflora* (Forssk.) R.Br. **8***Anubiasbarteri* Schott **9***Cyperusrheophyticus* Lye **10**CyperustonkinensisC.B.Clarkevar.baikiei (C.B.Clarke ex Kuk) S.S.Hooper.

Cameroon (Fig. 2), Democratic Republic of Congo, Gabon, Ghana, and Liberia.

####### Conservation status in Cameroon.

*Bolbitisfluviatilis* is not listed on http://www.iucnredlist.org nor in Onana and Cheek (2011). The species is currently known from five localities. The extent of occurrence (EOO) is more than 20,000 km^2^, and the area of occupancy (AOO) is about 20 km^2^. Some collecting localities of the species are within the Cameroon Development Corporation (CDC) palm tree plantations, and other localities are proposed for plantation development. Based on this threat, extent and/or quality of the habitat of the species *B.fluviatilis* is here assessed as Endangered. IUCN Red List Category: **Endangered ENB2ab (ii, iii).**

###### Bolbitis heudelotii

Taxon classificationPlantaePolypodialesLomariopsidaceae

(Bory ex Fée) Alston, J. Bot. 72 (Suppl. 2): 3 (1934)

Gymnopteris heudelotii Bory ex Fée, Mém. Foug., 2. Hist. Acrostich. 84: 45, t. 45 (1845)
Leptochilus heudelotii (Bory ex Fée) C.Chr., Index Filic. 11, 385 (1905)

####### Type.

Guinea Conakry, Fouta Djallon, in herb Bory, *Heudelot 803* (holotype: P).

####### Description.

Herbaceous, rhizome thick with dark brown scales and creeping on rocks; numerous roots; sterile fronds 30–80 cm long, linear to elliptical; fertile fronds 25–100 cm, long, linear, abaxial surface with sporangia.

####### Specimens examined.

Pangar River, at Tapare (Dang Assoura), 5°22'N, 13°31'E, 11 Feb 1961, *R. Letouzey 3452* (YA); Maan, 24 km southeast of Nyabesan, 00 Feb 1963, *J. & A*. *Raynal 10264* (YA); East of Kribi on Kienke River, 2°56'N, 9°55'E, 05 Apr 1969, *J. J. Bos 4282* (YA); Limbe at Limbe River, 4°2'N, 9°12'E, 13 Jan 2011, *F. Kuetegue 412, 414* (YA).

####### Habitat.

Riverbeds of perennial streams; edge of waterfalls; seasonally flooded in swift-flowing rivers or streams, able to withstand spate; in rainforest.

####### Distribution.

Widespread in tropical Africa. Angola, Benin, Cameroon (Fig. 3), Central African Republic, Congo, Côte d’Ivoire, Democratic Republic of Congo, Ghana, Guinea, Malawi, Mali, Mozambique, Nigeria, Sierra Leone, Tanzania, Togo, Zambia, and Zimbabwe.

####### Conservation status in Cameroon.

*Bolbitisheudelotii* is not listed on http://www.iucnredlist.org nor in Onana and Cheek (2011). The extent of occurrence of this species is about 102,900 km^2^ with an area of occupancy of about 32 km^2^. The species is currently known from 8 localities. The main pressures on the habitats are logging; dam construction (i.e. Lom-Pangar Hydro-electric dam, between Lom and Pangar) and expansion of cocoa farms. Based on these threats, extent and/or quality of habitat *B.heudelotii* is here assessed as Vulnerable. IUCN Red List Category: **Vulnerable VUB2ab (ii, iii).**

##### Acanthaceae

###### Lepidagathis alopecuroides

Taxon classificationPlantaeLamialesAcanthaceae

R.Br. ex Griseb., Fl. Brit. W. I. 453 (1862)

Adenosma chenopodiifolia (Poir.) Spreng., Syst. Veg. ed. 16, 2: 829 (1825)
Aetheilema alopecuroidea (Vahl) Spreng., Syst. Veg. 2: 826 (1825)
Ruellia alopecuroidea Vahl, Eclog. Amer. 2: 49 (1798)
Ruellia chenopodiifolia Poir., Encycl. 6(1): 339 (1804)
Teliostachya alopecuroidea Nees, Prodr. 11: 263 (1847)

####### Type.

Sierra Leone, by Scaries River, 1891, *G. F. Scott Elliot 4533* (K, K000529239).

####### Description.

Herb, slender, flexible, decumbent and branching stems, with lanceolate leaves 5.0–6.5 × 0.4–0.6 cm; strong fibrous root system; pink or purplish flowers.

####### Specimens examined.

40 km northwest of Moloundou on Dja River, 18 Mar 1973, *R. Letouzey 12132* (YA); Ndian 50 m on bank of Mana River, 4°58'N, 8°51'E, 09 Dec 1983, *D. W. Thomas 2659* (YA); Mundemba on Mana River, 11 Jan 1998, *M. Cheek 8850* (YA).

####### Habitat.

Rocky and sandy riverbeds or on the banks of rivers and streams; in rainforest.

####### Distribution.

Benin, Cameroon (Fig. 4), Côte d’Ivoire, Gabon, Ghana, Guinea and Nigeria.

####### Conservation status in Cameroon.

*Lepidagathisalopecuroides* is not listed on http://www.iucnredlist.org nor in Onana and Cheek (2011). The extent of occurrence of this species is estimated at 124,600 km^2^ and has an area of occupancy of about 20 km^2^. The taxon is currently known from five localities. Construction of dams are in progress at two sites for this species: a hydroelectric dam at Natchigal on the Sanaga River; and another on the Dja River, and if fully operational the habitat of *L.alopecuroides* may be destroyed. Based on these threats, and the fact that the species is only known from five localities, *L.alopecuroides* is here assessed as Endangered. IUCN Red List Category: **Endangered ENB2ab (ii, iii).**

##### Amaranthaceae

###### Achyranthes talbotii

Taxon classificationPlantaeCaryophyllalesAmaranthaceae

Hutch. & Dalziel, Fl. W. Trop. Afr. 1: 127 (1927)

####### Type.

Nigeria, *Keay, R. W. J. FHIFHI 28284* (holotype: K, K000243718).

####### Description.

Perennial herb with soft woody stem to 45 cm tall; strong fibrous root system; lanceolate leaves 2–4 × 1–1.5 cm.

####### Specimens examined.

Near Ndokman II, approximately 8 km east of Yingui or 35 km east of Yabassi, 4°34'N, 10°10'E, 00 Jan 1972, *R. Letouzey 10938* (YA); bank of Nkam River, near Sake, 3 km southwest of Nkondjok, 4°77'N, 10°17'E, 07 Jan 1972, *R. Letouzey 11163* (YA); Mumgo River, Kumba-Loum road, 2°49'N, 9°33'E, 00 Jan 1981, *Breyne 5062* (YA); Kombon at the bank of Kombon River, 4°59'N, 9°26'E, 23 Mar 2011, *F. Kuetegue 316* (YA).

####### Habitat.

Sandy and rocky riverbeds or up to flood level on the bank, in rainforest.

####### Distribution.

Cameroon (Fig. 5) and Nigeria.

####### Conservation status in Cameroon.

*Achyranthestalbotii* was assessed by Cheek (2014) globally as Near Threatened (NT) at http://www.iucnredlist.org. The species was, however, assessed by Onana and Cheek (2011) for Cameroon as Vulnerable, since at that time it was known from only 10 sites. The taxon is currently known from about 25 localities. The extent of occurrence is estimated to be above 20,000 km^2^ and the area of occupancy is about 100 km^2^. At some localities, road construction and arable farming are in progress but on a limited scale. Although the habitat of the species is under pressure, it does not appear to qualify as threatened under the IUCN red list criteria (IUCN 2013, 2017). While the expectation is that human pressure will increase the loss of habitat, and reduce the area of occupancy and extent of occurrence, it is not anticipated these threats will be significant. *A.talbotii* is here reassessed as of Least Concern. IUCN Red List Category: **Least Concern (LC).**

##### Amaryllidaceae

###### Crinum natans

Taxon classificationPlantaeAsparagalesAmaryllidaceae

Baker., Fl. Trop. Afr. 7 (3): 396 (1898)

Crinum natans Baker subsp. inundatum Kwembeya & Nordal; Phylogeny Speciation Biogeogr. Crinum Chlorophytum 3:16 (2008)

####### Type.

Equatorial Guinea, Fernando Po (Bioko), *G. Mann 1416* (lectotype: K; isotype: P).

####### Description.

Herb with small bulb, very strong root system; leaves crinkled, submerged and floating, 140 × 2.2 cm; flowers large, borne above the water.

####### Specimens examined.

15 km north of Edea, near the bridge, 22 Jan 1969, *J. J. Bos 1969* (YA); Balondo, 25 km southwest of Nkongsamba, 4°43'N, 9°51'E, 00 Mar 1976, *R. Letouzey 14441* (YA); Soo village, near bridge on Soo River, 3°20'N, 11°30'E, 06 Apr 1977, *Inger Nordal 906* (YA); Diongo (Kumba – Nguti road) on bank of Mengue River, 4°45'N, 9°29'E, 21 Mar 2011, *F. Kuetegue 507* (YA).

####### Habitat.

Bed of swift-flowing perennial streams and rivers, submerged permanently, strong fibrous root system, in sand, silt or gravel riverbeds; in evergreen rainforest.

####### Distribution.

Cameroon (Fig. 6), Côte d’Ivoire, Gabon, Ghana, Guinea, Liberia, Nigeria, and Sierra Leone.

####### Conservation status in Cameroon.

*Crinumnatans* was not listed on http://www.iucnredlist.org nor assessed by Onana and Cheek (2011). The taxon is currently known from 10 localities. The extent of occurrence of *C.natans* is about 92,850 km^2^ and the area of occupancy is about 40 km^2^. Human activities at the localities include timber exploitation; and planned mining operations, hydroelectric dams and plantation development. Based on these threats, and the continuous decline of the vegetation cover in the area, extent and/or quality of its habitat, the species is here assessed as Vulnerable. IUCN Red List Category: **Vulnerable VUB2ab (ii, iii).**

##### Asclepiadaceae

###### Kanahia laniflora

Taxon classificationPlantaeGentianalesAsclepiadaceae

(Forssk.) R.Br., Voy. Abyss. App.: Ixiv (1814)

Asclepias coarctata S.More, J. Bot. 46: 297 (1908)
Asclepias fluviatilis A.Chev., Bull. Soc. Bot. France 61(8): 271 (1917)
Asclepias laniflora Forssk., Fl. Aegypt.-Arab. 51 (1775)
Asclepias rivalis S.More, J. Bot. 52: 337 (1914)
Gomphocarpus glaberrimus Oliv., Trans. Linn. Soc. London 29(3): 110 (1875)
Kanahia consimilis N.E.Br., Fl. Trop. Afr. 4(1.2): 298 (1902)
Kanahia glaberrima (Oliv.) N.E.Br., Fl. Trop. Afr. 4(1.2): 297 (1902)

####### Type.

Cameroon, *G. L. Bates 322* (lectotype: K, K000234855).

####### Description.

Erect woody shrub, up to 2 m high; leaves linear-lanceolate 4–15 × 0.5–1.0 cm, glabrous; inflorescence axillary, one per node between leaf bases; flowers creamy white.

####### Specimens examined.

Dibombe River, 4°41'N, 9°48'E, 16 Mar 1965, *A. J. M. Leeuwenberg 9708* (YA); bank of UVE River, 20 km northwest of Kumba, 20 Mar 1976, *R. Letouzey 38378* (YA); Mundemba, on Ndian (Mana) River, 4°58'N, 8°51'E, 09 Dec 1983, *D. W. Thomas* 2656 (YA); Ntale, bank and bed of Mbier River, 10 Dec 2010, *F. Kuetegue 272, 273* (YA).

####### Habitat.

Rocky or sandy riverbeds and by seasonal rivers, in wet evergreen and semi-deciduous rainforests to deserts.

####### Distribution.

Benin, Cameroon (Fig. 7), Côte d’Ivoire, Nigeria, Somalia, South Africa, and Sudan.

####### Conservation status in Cameroon.

*Kanahialaniflora* was not assessed by Onana and Cheek (2011). It is listed on http://www.redlist.org as Least Concern (LC) by Lansdown (2013). This is because the species, globally, is believed to be widespread and abundant throughout much of its known distribution area. The species is distributed from Saudi Arabia and Yemen, through Ethiopia and Somalia to Cote d’Ivoire and South Africa. The extent of occurrence of *K.laniflora* in Cameroon is about 78,700 km^2^; and area of occupancy of the species is about 32 km^2^. The taxon is currently known from 8 localities in the country. The localities where the species is found are proposed for timber exploitation and plantation development. Based on these threats and the progressive destruction of the vegetation in the localities, extent and/or quality of the habitats, the species is here assessed, as Vulnerable. IUCN Red List Category: **Vulnerable VUB2ab (ii, iii).**

##### Araceae

###### Anubias barteri

Taxon classificationPlantaeAlismatalesAraceae

Schott, Prodr. Syst. Aroid. 159 (1860)

####### Type.

Equatorial Guinea, Fernando Po (Bioko), *Barter 2045* (K).

####### Description.

Hardy herb with thick creeping rhizome, prostrate; strongly rooted; green, lush, narrow shaped leaves 7–30 × 3–15 cm.

####### Specimens examined.

Fenda, 60 km southeast of Kribi, 22 Jan 1962, *R. Letouzey 4120* (YA); Nyon River, 49 km southwest of Eseka, 12 Mar 1965, *A. J. M. Leeuwenberg 5136* (YA); road from Ebone to Yabassi at mile 10, 27 Dec 1967, *P. Bamps 1636* (YA), Nguti-Kombon, bank of Kombon River, 5°13'N, 9°33'E, 13 Dec 2010, *F. Kuetegue 239* (YA).

####### Habitat.

Rocky beds of swift-flowing streams and rivers, or wet shrubby and bushy bank of rivers and streams; in rainforest.

####### Distribution.

Cameroon (Fig. 8), Congo, Côte d’Ivoire, Gabon, Guinea, Liberia, and Nigeria.

####### Conservation status in Cameroon.

*Anubiasbarteri* is listed on http://www.iucnredlist.org as Least Concern (LC) in central Africa in 2007 by Ghogue (2010a). The species was not assessed by Onana and Cheek (2011). The taxon is currently known from 16 localities. The extent of occurrence of *A.barteri* is about 7,600 km^2^ and has an area of occupancy of about 64 km^2^. The habitat is mainly threatened by urban development, road constructions and hydroelectric dams already built or at project stage. Despite the threats, and the fact that the habitats are under pressure, the species does not appear to qualify as threatened under the IUCN red list criterion (IUCN 2017). Though human pressure is expected to increase the loss of habitat and reduce area of occupancy and extent of occurrence (EOO), it is not expected that this will be significant. It is possible that the EOO was underestimated because only specimens with geographical coordinates were used to estimate the EOO. Based on these observations, *A.barteri* is assessed here as Near Threatened. IUCN Red List Category: **Near Threatened (NT).**

##### Cyperaceae

###### Cyperus rheophyticus

Taxon classificationPlantaePoalesCyperaceae

Lye, Nordic J. Bot. 24 (3): 273 (2006)

####### Type.

Cameroon, South West Division, Kupe-Muanenguba Division, Muambong, bank of Chide River, 3°58'N, 9°41'E, 02 Aug 1998, *J.-M. Onana 585*, (holotype: K; isotype: YA).

####### Description.

Perennial herb, 30–50 cm high; deeply rooted; inflorescence terminal, with involucre bracts.

####### Specimens examined.

Kodmin, beside a stream, 4°59'N, 9°42'E, 21 Nov 1998, *M. Etuge 406* (YA).

####### Habitat.

Forest streams and rivers, submerged during wet season.

####### Distribution.

Cameroon (Fig. 9).

####### Conservation status in Cameroon.

*Cyperusrheophyticus* is listed on http://www.iucnredlist.org as Vulnerable in 2017 (Cheek et al. 2017b). The taxon is currently known from five localities, and endemic to Cameroon. The extent of occurrence of this species is about 3,000 km^2^ and area of occupancy is about 20 km^2^. The localities where they occur are proposed for plantation development, timber exploitation and road construction. Not much has changed since the last assessment of Cheek et al. (2017b); here we maintain its status as Vulnerable. IUCN Red List Category: **Vulnerable B1ab (iii) +2ab (iii).**

###### Cyperus tonkinensis C.B.Clarke var. baikiei

Taxon classificationPlantaePoalesCyperaceae

(C.B.Clarke ex Kük) S.S.Hooper, Kew Bull. 26 (3): 577 (1972)

Cyperus baikiei C.B.Clarke, Consp. Fl. Afr. [T.A. Durand & H. Schinz] 5: 550 (1894)
Cyperus kottensis Chem. Arch. Bot., Caen iv. Mem. No. 7, 23 (1931)

####### Type.

Vietnam, Tonkin, Tu-Phap, 12 Apr 1888, *Balansa 2831* (K).

####### Description.

Herb, about 12 cm high, with hard tubers, connected by dark brown rhizome; stems dark brown and shiny; inflorescence spikelets, glumes brown.

####### Specimens examined.

62 km southeast of Bafia, on Sanaga River, 4°75'N, 11°22'E, 27 Mar 1963, *J. & A. Raynal 10538* (YA); Sanaga River, bridge near Nkong Njok, 4°10'N, 11°01'E, 12 Mar 1978, *J. Lowe 3483* (YA); Bongossi Research Plot of the National Herbarium of Cameroon, bank of Sanaga River, 4°22'N, 11°16'E, 29 Mar 1987, *L. Ake Asse 1859* (YA); near Nguti, Mbombe on Loa River, 29 May 2011, *F. Kuetegue, 498* (YA).

####### Habitat.

Sandy bars or beds of streams and rivers.

####### Distribution.

Benin, Cameroon (Fig. 10), Central African Republic, Chad, Congo, Ghana, Guinea, Liberia, Mali, Nigeria, Senegal, and Sierra Leone.

####### Conservation status in Cameroon.

Cyperustonkinensisvar.baikiei is not listed on http://www.iucnredlist.org nor assessed by Onana and Cheek (2011). The taxon is currently known from five localities. The extent of occurrence of this species is about 800 km^2^ and the area of occupancy is about 20 km^2^. Construction of a hydropower dam on the Sanaga River from which the species was collected is in progress. Based on this threat and the fact that the species is only currently known from five localities, C.tonkinensisvar.baikiei is here assessed as Endangered. IUCN Red List Category: **Endangered ENB1+2ab (ii, iii)**.

###### Cyperus cataractarum

Taxon classificationPlantaePoalesCyperaceae

K.Schum ex. Engl., Veg. Erde 9(2): 200 (1908)

Pycreus cataractarum C.B.Clarke, Bot. Jahrb. Syst. 38(2): 132 (1906)

####### Type.

Cameroon, Bipindi, 1899, *G. Zenker 1935* (syntype: BR, K, P, P00573020, WAG).

####### Description.

Tufted, grass-like leaves forming high sods (up to 30 cm thick cushions); roots forming large fibrous tussocks; stems smoothly glossy; leaves linear, green to dark green; spikelets greenish-white; glumes with a green midrib.

####### Specimens examined.

27 km from Kribi in Kienké River, 2°52'N, 10°7'E, 27 Jan 1970, *J. J. Bos 6168* (YA); Mpoume waterfalls on Nyong River at Makak, 3°28'N, 11°01'E, 01 Jan 1978, *J. Lowe 3420* (YA); near Akonetyè village, Mboro waterfalls, 15 Jan 1978, *A. Koufani 20* (YA).

####### Habitat.

Banks, edges and beds of streams and rivers; in rainforest.

####### Distribution.

**Figures 11–19.**
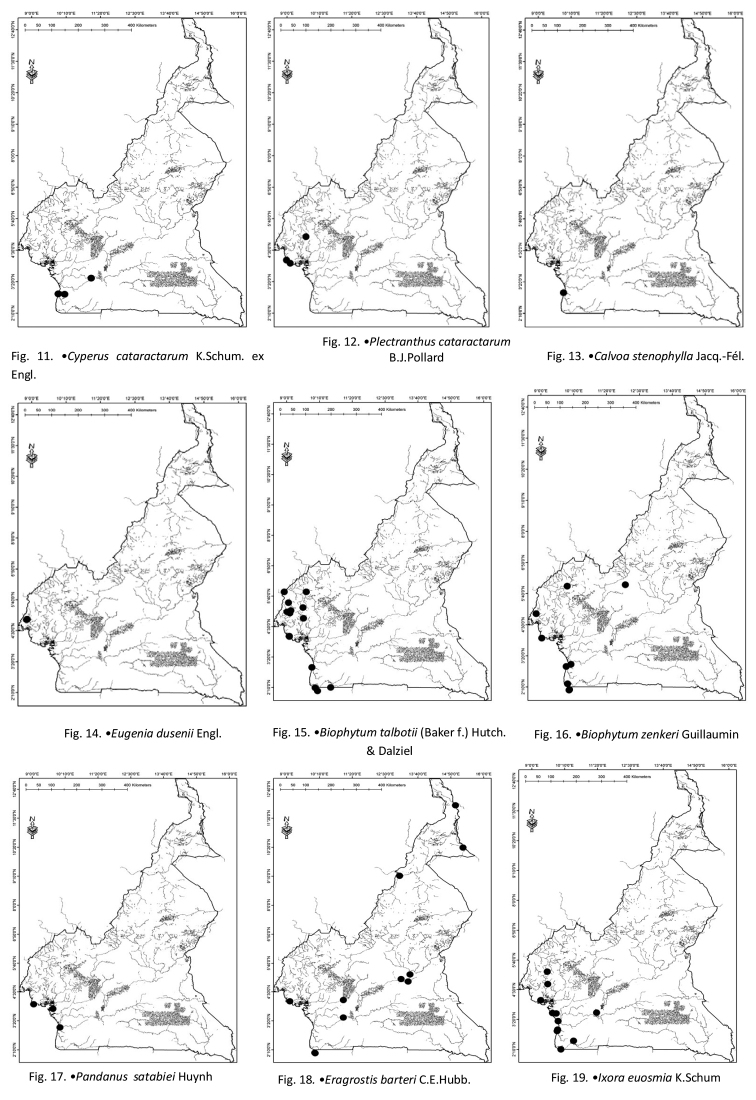
**11***Cyperuscataractarum* K.Schum ex Engl. **12***Plectranthuscataractarum* B.J.Pollard **13***Calvoastenophylla* Jacq.-Fél. **14***Eugeniadusenii* Engl. **15***Biophytumtalbotii* (Baker f.) Hutch. & Dalziel **16***Biophytumzenkeri* Guillaumin **17***Pandanussatabiei* Huynh **18***Eragrostisbarteri* C.E.Hubb. **19***Ixoraeuosmia* K.Schum.

Cameroon (Fig. 11), Congo, Gabon and Nigeria.

####### Conservation status in Cameroon.

*Cyperuscataractarum* is not listed on http://www.iucnredlist.org nor assessed by Onana and Cheek (2011). The taxon is currently known from 6 localities. The extent of occurrence of this species is about 13,400 km^2^ and the area of occupancy is about 24 km^2^. The habitat of *C.cataractarum* is being progressively destroyed by plantation development and timber exploitation. Based on these threats, and extent and/or quality of habitat, the species is currently assessed as Vulnerable. IUCN Red List Category: **Vulnerable VUB1+2ab (ii, iii).**

##### Lamiaceae

###### Plectranthus cataractarum

Taxon classificationPlantaeLamialesLamiaceae

B.J.Pollard, Kew Bull 56(4): 976 (2001)

####### Type.

Cameroon: Hunters path to Lake Njonji at side of seasonal watercourse, *M. Cheek 5563* (holotype: K; isotypes: MA, MO, SCA, WAG, YA).

####### Description.

Annual or perennial herb growing to 60 cm tall; stems decumbent to ascending, sub-woody at the base; leaves slightly fleshy, 20–45(-70) × 5–20(-25) mm, 2–2.5 times as long as broad; inflorescence terminal.

####### Specimens examined.

Etinde, Njonji, footpath from Cameroon Development Corporation oil palm plantations to the summit, 24 Nov 1993, *Williams 52* (K, SCA, WAG, YA); Bakossi Mts: Chutes de ‘Ile Ndip Medschang, 21 Nov 1998, *Satabie 1109* (K, K000051130).

####### Habitat.

Invariably growing in spray zone of waterfalls, on wet rocks or on river banks, up to flood level, of swift-running water; lowland or submontane evergreen forest, 300–1450 m alt.

####### Distribution.

Cameroon (Fig. 12) and Equatorial Guinea.

####### Conservation status in Cameroon.

*Plectranthuscataractarum* is listed on http://www.iucnredlist.org. The species was assessed as Vulnerable (Pollard and Paton 2003). Eight years later it was reassessed as Endangered (Onana and Cheek 2011). The taxon is currently known from three collecting localities. The extent of occurrence and the area of occupancy are both estimated to be less than 10 km^2^. The associated threats, such as forest logging and plantation establishment, mentioned by Pollard and Paton (2003) are still ongoing. Based on these threats, and that the habitats are still under pressure, the species is here re-evaluated as Endangered. IUCN Red List Category: **Endangered ENB1+2ab (ii+iii).**

##### Melastomataceae

###### Calvoa stenophylla

Taxon classificationPlantaeMyrtalesMelastomataceae

Jacq.-Fél., Bull. Mus. Natl. Hist. Nat., B, Adansonia Sér. 4, 3(2): 143 (1981)

####### Type.

Cameroon, 10 km Southeast of Zingui, 16 Mar 1968, *R. Letouzey 9083*. (Holotype: P; isotype: YA).

####### Description.

Small herb, about 20 cm high; stem flexible; roots spreading and fibrous; leaves narrow-lanceolate 4–6 × 0.2–0.5 cm; flowers terminal, pink.

####### Specimen examined.

Minsomo River, 10 km southeast of Zingui, 2°56'N, 9°54'E, 16 Mar 1968, *R. Letouzey 9083* (YA).

####### Habitat.

Rocks on bed of Minsomo River.

####### Distribution.

Cameroon (Fig. 13) and Equatorial Guinea.

####### Conservation status in Cameroon.

*Calvoastenophylla* is listed on http://www.iucnredlist.org as Endangered (Cheek 2015). The species was assessed as Critically Endangered (Onana and Cheek 2011), since it was known from only one locality and the associated threats at the time. Cheek (2015) reassessed the species, globally as Endangered, since the number of localities has increased to two, and area of occupancy of 8 km^2^. The second locality is in Equatorial Guinea (Cheek 2015). In Cameroon, the species is only known from one locality and the area of occupancy is about 4 km^2^. The extent of occurrence (EOO) is estimated at 1 km^2^, following the IUCN preferred grid cell size for aquatic organisms (IUCN 2013, 2017). This was used by Cheek et al. (2015) in assessing the conservation status of *Ledermanniellalunda* Cheek (Podostemaceae) from Angola. The main threats are forest logging and agriculture activities. Based on these threats, and the continuous decline of vegetation in the area, extent and /or quality of habitat *C.stenophylla* is here assessed, as Critically Endangered. IUCN Red List Category: **Critically Endangered CRB2ab (ii, iii).**

##### Myrtaceae

###### Eugenia dusenii

Taxon classificationPlantaeMyrtalesMyrtaceae

Engl., Notizbl. Königl. Bot. Gart. Berlin 2: 289 (1899)

Myrtus dusenii Kuntze, Deutsche Bot. Monatsschr. 21: 173 (1903)

####### Type.

Cameroon. Mundemba, Mana bridge, 4°58'N, 7°00'E, 11 Jan 1998, *M. Cheek 8845* (holotype: K; isotype: YA).

####### Description.

Small erect shrub, up to 1.5 m; stem very flexible; leaves small and narrow, 2–5 × 0.3–0.5 cm; strongly rooted; white-flowered.

####### Specimens examined.

Ndian waterfalls at Bulu docks, 4°56'N, 8°51'E, 17 Jan 1985, *D. W. Thomas 1985* (YA); Ndian River, west of Mundemba, 5°02'N, 8°53'E, 21 Jan 1986, *J. Nemba & D. W. Thomas 319* (YA); Mundemba, Mana bridge, 4°58'N, 7°00'E, 11 Jan 1998, *M. Cheek 8845* (YA).

####### Habitat.

Beds of swift-running rivers; seasonally inundated river banks; rocks at waterfalls, in evergreen rainforest.

####### Distribution.

Cameroon (Fig. 14), endemic to Cameroon.

####### Conservation status in Cameroon.

*Eugeniadusenii* is not listed on http://www.iucnredlist.org, but it was assessed as Vulnerable VU in Onana and Cheek (2011). The taxon is endemic to Cameroon and currently known from four localities. The area of occupancy is estimated to be about 16 km^2^, and the extent of occurrence is estimated at 4 km^2^. Plantation development and illegal logging of timber are ongoing at the localities. Based on these threats, and the continuous decline of vegetation cover in the area, and extent and /or quality of habitat *E.dusenii* is currently reassessed as Endangered. IUCN Red List Category: **Endangered ENB2ab (ii, iii).**

##### Oxalidaceae

###### Biophytum talbotii

Taxon classificationPlantaeOxalidalesOxalidaceae

(Baker f.) Hutch. & Dalziel, Fl. W. Trop. Afr. 1: 140 (1927)

Biophytum kamerunense Engl. & R.Kunth ex Engl., Veg. Erde 9(3,1): 717 (1915)
Oxalis talbotii Baker f., Cat. Pl. Oban 16 (1913)

####### Type.

Liberia, 02 Nov 1910, *Bunting, R. H. 103* (holotype: BM).

####### Description.

Perennial herb, woody stem, up to 30 cm high; roots spreading; leaves in shape of umbrella; flowers pink.

####### Specimens examined.

Njabilobé, 54 km southeast of Kribi, 12 Mar 1963, *J. & A. Raynal 10425* (YA); Kienke River, Kribi, 2°56'N, 9°55'E, 20 Jun 1969, *J. J. Boss 4900* (YA); near Numba, 45 km northeast of Mamfe, 5°50'N, 9°42'E, 18 Aug 1975, *R. Letouzey 14331* (YA); Mamfe road, near Numba, 16 Dec 2012, *F. Kuetegue 400* (YA) .

####### Habitat.

Riverbeds and earthbanks of shaded forest streams, periodically inundated rocks in rivers; in rainforest.

####### Distribution.

Cameroon (Fig. 15), Liberia and Nigeria.

####### Conservation status in Cameroon.

*Biophytumtalbotii* is not listed on http://www.iucnredlist.org nor assessed by Onana and Cheek (2011). The taxon is currently known from 13 localities. Extent of occurrence of this species is about 38,000 km^2^ and the area of occupancy is about 56 km^2^. Forest logging is the main threat at these habitats. Based on this threat and the continuous decline of vegetation cover in the area, extent and /or quality of habitat *B.talbotii* is here assessed as Vulnerable. IUCN Red List Category: **Vulnerable VU B2ab (ii, iii).**

###### Biophytum zenkeri

Taxon classificationPlantaeOxalidalesOxalidaceae

Guillaumin, Notul. Syst. (Paris) 1: 26 (1909)

####### Type.

Cameroon, 01 Jan 1908, *G. Zenker 3428* (BM, BR, G; HBG, K, K000419376, M, P, W).

####### Description.

Perennial herb, up to 30 cm tall, forming dense clumps; leaves in rosette or nearly so; flowers yellow.

####### Specimens examined.

Cross River ferry between Ikom and Manfe, 07 Apr 1955, *J. K. Morton K318* (YA); Korup, rocky river bank of Mana River, 4°55'N, 8°50'E, 08 Jun 1983, *D. W. Thomas 2164* (YA); Ndian Division Mundemba, in Mana River, 5°00'N, 8°50’E, 21 Nov 1986, *Stephen D. Manning 896* (YA).

####### Habitat.

In rock crevices in riverbeds, seasonally flooded; in forest.

####### Distribution.

Angola, Cameroon (Fig. 16), Congo, Gabon and Nigeria.

####### Conservation status in Cameroon.

Like the species before it, *Biophytumzenkeri* is not listed on http://www.iucnredlist.org. The taxon is currently known from 9 localities. The extent of occurrence of this species is about 80,000 km^2^ and its area of occupancy is about 36 km^2^. Plantation development is in progress at two of the localities and this may affect the survival of the species. Based on this threat, and the continuous decline of vegetation cover in the area, extent and /or quality of habitat *B.zenkeri* is here assessed as Vulnerable. IUCN Red List Category: **Vulnerable VU B2ab (ii, iii).**

##### Pandanaceae

###### Pandanus satabiei

Taxon classificationPlantaePandanalesPandanaceae

Huynh, Bull. Mus. Natl. Hist. Nat., B, Adansonia Sér. 4, 6(3): 347 (1984)

####### Type.

Cameroon, near the Ndonga River (30 km W Edea), 20 Dec 1973, *R. Letouzey 12472* (holotype: P; isotype: YA).

####### Description.

Shrub or small tree of about 5 m tall; strongly rooted; leaves narrow 60–80 × 2–4 cm, spine on the borders; fruit green.

####### Specimens examined.

Wouri River, near Bekoko, Douala-Nkongsamba road, 16 Jun 1983, *Satabie 674* (YA); bed of Dilolo River at Bolomeboka, Nkonyé, 4°51'N, 9°28'E, 22 Mar 2011, *F. Kuetegue 462* (YA); Mongo River at Mbakwa Super, Nkonyè, 5°01'N, 9°25'E, 24 Mar 2011, *F. Kuetegue 463* (YA).

####### Habitat.

Banks of rivers subject to flooding.

####### Distribution.

Cameroon (Fig. 17).

####### Conservation status in Cameroon.

*Pandanussatabiei* was not assessed by Onana and Cheek (2011) nor listed on http://www.iucnredlist.org. The taxon is endemic to Cameroon and currently known from five localities. The extent of occurrence of this species is about 2,900 km^2^ and the area of occupancy is about 20 km^2^. Human settlements are developing around one collecting locality; also arable farming is in progress along the rivers. Based on these threats, and the continuous decline of vegetation cover in the area, extentand /or quality of habitat, *P.satabiei* is here assessed as Endangered. IUCN Red List Category: **Endangered EN B1+2ab (ii, iii).**

##### Poaceae

###### Eragrostis barteri

Taxon classificationPlantaePoalesPoaceae

C.E.Hubb., Fl. W. Trop. Afr. 2: 514 (1936)

Eragrostis fluviatilis A.Chev., Bull. Mus. Nalt. Hist. Nat. sér 2, 20: 472 (1948)

####### Type.

Nigeria, 1858, *C. Barter 877* (syntype: K, K000366508; isotype: P).

####### Description.

Perennial grass, robust, of about 1 m high; leaves lanceolate; inflorescence in open panicle.

####### Specimens examined.

Nkokmen II, at 8 km east of Yingui, at the bank of Makombe River, 4°32'N, 10°15'E, 09 Jan 1972, *D. van der Zon 10939* (YA). Mpoumé falls on Nyong River, 9 km south of Makak 3°28'N, 11°00'E, 20 Jan 1977, *J. Lowe 3188* (YA); Sanaga River at Nkongnjok, 4°10'N, 11°01'E, 12 Jan 1978, *J. Lowe 3471* (YA); Kikot (Douala-Bafia road), near bridge, bank of Sanaga, 3°51'N, 11°30'E, 16 Dec 2012, *F. Kuetegue 535* (YA).

####### Habitat.

Among rocks, and sandbanks on riverbed or streams.

####### Distribution.

Cameroon (Fig. 18), Congo, Côte d’Ivoire, Ghana, Mali, Nigeria, Senegal and Sierra Leone.

####### Conservation status in Cameroon.

*Eragrostisbarteri* was not assessed by Onana and Cheek (2011), nor listed on http://www.iucnredlist.org. The taxon is currently known from 10 localities some of which are on the Sanaga River. The extent of occurrence of the species is about 260,000 km^2^ and area of occupancy is about 44 km^2^. The proposed dam on Sanaga River will destroy some habitats of the species. Based on this threat, and the continuous decline of vegetation cover in the area, extent and /or quality of habitat, *E.barteri* is currently assessed as Vulnerable. IUCN Red List Category: **Vulnerable VU B2ab (ii, iii).**

##### Rubiaceae

###### Ixora euosmia

Taxon classificationPlantaeGentianalesRubiaceae

K.Schum., Bot. Jahrb. Syst. 33(2): 355 (1903)

Ixora degemensis Hutch. & Dalziel, Fl. W. Trop. Afr. 2: 86 (1931)

####### Type.

Cameroon. Bipindi, bank of Lokoundje River, 02 Oct 1896, *G. Zenker 1108*, (holotype: K; isotypes: BR, HBG, MO, WAG, Z).

####### Description.

Shrub or small tree up to 5 m tall; leaves narrowly elliptic-oblong 10–19 × 2–4.5 cm; inflorescence terminal.

####### Specimens examined.

Lobe River, 7 km south of Kribi, 2°53'N, 9°54'E, 20 Feb 1969, *J. J. Bos 3940* (YA); Songloulou falls, at 25 km southwest of Ngambé, Massock-Songloulou road, 3°35'N, 9°44'E, 24 Jan 1972, *R. Letouzey 11103* (YA); 5 km southeast of Bipindi, 3°04'N, 10°25'E, 14 Jan 1987, *Stephen D. Manning 1343* (YA); Nguti-Ntalè, Mbièr River, 5°15'N, 9°34'E, 13 Dec 2010, *F. Kuetegue 226* (YA).

####### Habitat.

Inundated sandy or rocky banks of rivers, between rocks in streams or rivers, waterfalls; riverine forest.

####### Distribution.

Cameroon (Fig. 19), Equatorial Guinea and Nigeria.

####### Conservation status in Cameroon.

*Ixoraeuosmia* was not assessed by Onana and Cheek (2011) nor listed on http://www.iucnredlist.org. The taxon is currently known from 10 localities. The extent of occurrence of this species is about 36,900 km^2^ and the area of occupancy is about 44 km^2^. There is a dam built (hydro-electric dam at Songloulou on Sanaga River), and a proposed project, Memve’ele hydro-electric dam at Nyabezan (Ntem waterfall); and mining projects envisaged in Kribi. Based on these threats and the continuous decline of vegetation cover in the area, extent and /or quality of habitat *I.euosmia* is here assessed as Endangered. IUCN Red List Category: **Endangered ENB2ab (ii, iii).**

###### Ixora inundata

Taxon classificationPlantaeGentianalesRubiaceae

Hiern, Fl. Trop. Afr. 3: 166 (1877)

####### Type.

Gabon. Cristal Mountains, 1862, *Mann 1731* (holotype: K; isotype: P).

####### Description.

Shrub up to 1.5 m tall; strongly rooted; stems tough; leaves lanceolate 4–11 × 1–2.5 cm; flowers translucent white.

####### Specimens examined.

Near Nkolemvom, 20 km southeast of d’Ebolowa, 2°54'N, 11°09'E, 03 Mar 1970, *R. Letouzey 9986* (YA); between Bulu and Ekum Bako, SW Region, 4°56'N, 8°52'E, 01 Jun 1984, *D. W. Thomas 3499* (YA); at the bank of Cross River, north of Nsanaragati, 5°52'N, 8°54'E, 16 Dec 1986, *Stephen D. Manning 1217* (YA).

####### Habitat.

Rocky bank of rivers, periodically inundated; between rocks in rivers.

####### Distribution.

**Figures 20–25.**
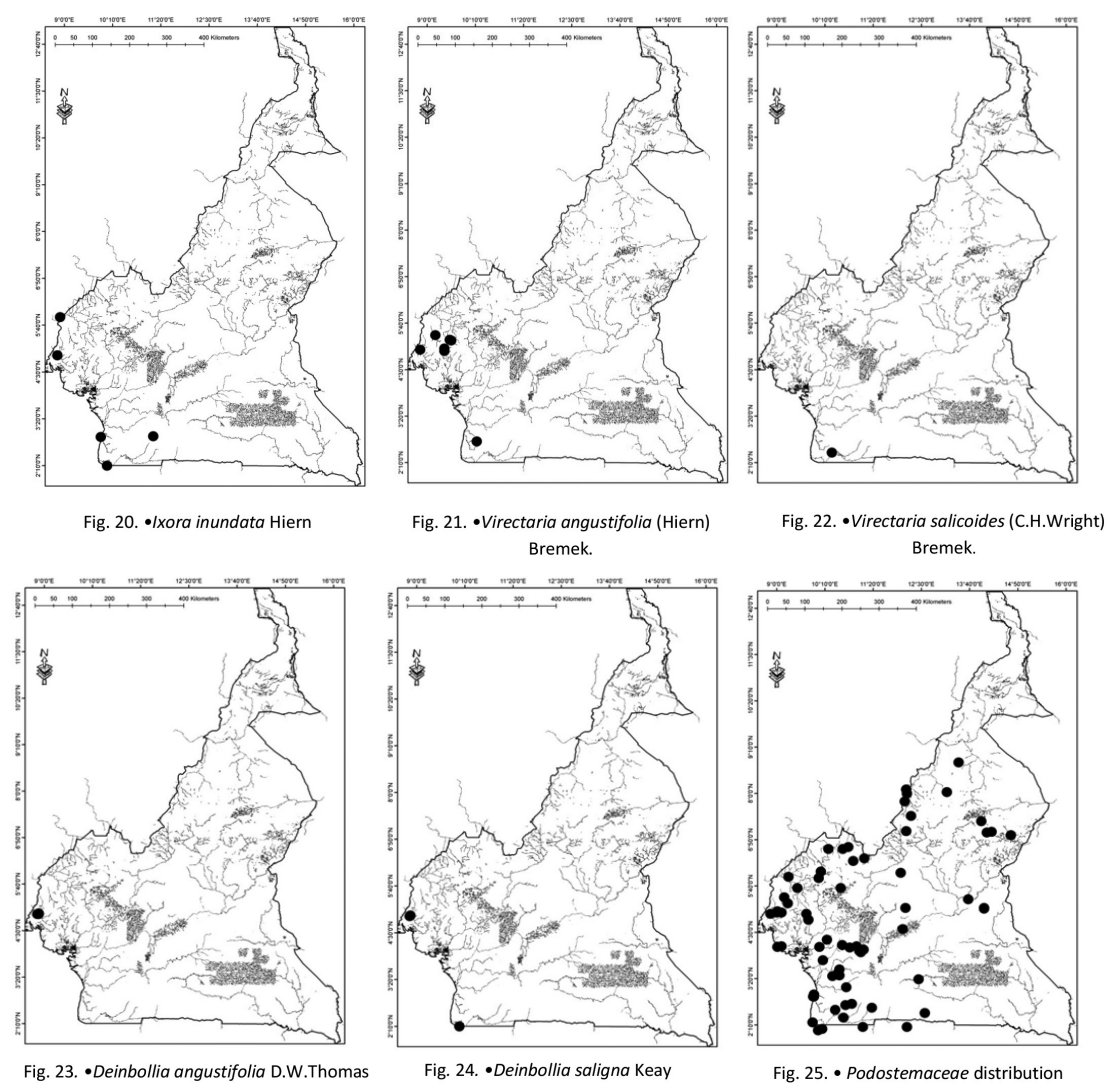
**20***Ixorainundata* Hiern **21***Virectariaangustifolia* (Hiern) Bremek **22***Virectariasalicoides* (C.H.Wright) Bremek **23***Deinbolliaangustifolia* D.W.Thomas **24***Deinbolliasaligna* Keay **25***Podostemaceae* distribution.

Cameroon (Fig. 20) and Gabon.

####### Conservation status in Cameroon.

*Ixorainundata* is not listed on http://www.iucnredlist.org. However, in Onana and Cheek (2011), the species was assessed as Endangered. The taxon is currently known from five localities. The extent of occurrence of the species is about 56,000 km^2^, and the area of occupancy is about 20 km^2^. The proposed dam on Ntem River is very likely to threaten the survival of *I.inundata*. Based on this threat and the continuous decline of vegetation cover in the area, extent and /or quality of habitat the species is assessed here as Endangered. IUCN Red List Category: **Endangered ENB2ab (ii, iii).**

###### Virectaria angustifolia

Taxon classificationPlantaeGentianalesRubiaceae

(Hiern) Bremek., Verh. Kon. Ned. Akad. Wetensch, Afd. Natuurk., Sect. 2. 48(2): 21 (1952)

Virecta angustifolia Hiern, Fl. Trop. Afr. 3: 48 (1877)
Virecta heteromera K.Schum., Bot. Jahrb. Syst. 23(3): 422 (1896)
Virectaria heteromera (K.Schum.) Bremek., Verh. Kon. Ned. Akad. Wetensch., Afd. Natauurk., Sect. 2, 48(2): 21 (1952).

####### Type.

Gabon, Mounts of Cristal, July 1862, *G. Mann 1686* (holotype: K).

####### Description.

Erect herb, up to 30 cm tall; leaves linear to oblanceolate 5–15 × 1–2 mm; flowers white.

####### Specimens examined.

50 km southeast of Kribi, 2°42'N, 10°12'E, 14 Mar 1968, *R. Letouzey 9011* (YA); bank of Mana River at Ndian, 4°58'N, 8°51'E, 09 Dec 1983, *D. W. Thomas 50588* (YA); Ndian River northwest of Mundemba, 00 Oct 1986, *Stephen D. Manning 894* (YA); Ntale, bank and bed of Mbier and Essembe River, 23 Dec 2010, *F. Kuetegue 319* (YA).

####### Habitat.

Rocks of riverbeds and inundated banks, up to flood level of streams and rivers, submerged during rainy season.

####### Distribution.

Cameroon (Fig. 21), Gabon, Ghana and Nigeria.

####### Conservation status in Cameroon.

*Virectariaangustifolia* is not listed on http://www.iucnredlist.org, nor assessed by Onana and Cheek (2011). The taxon is currently known from more than 7 localities. The extent of occurrence of the species is about 66,500 km^2^, and its area of occupancy is about 28 km^2^. Timber exploitation and plantation development are in progress at the collecting localities. Based on these threats and the continuous decline of vegetation cover in the area, extent and /or quality of habitat *V.angustifolia* is here assessed as Vulnerable. IUCN Red List Category: **Vulnerable VB1+2ab (ii, iii).**

###### Virectaria salicoides

Taxon classificationPlantaeGentianalesRubiaceae

(C.H.Wright) Bremek., Verh. Kon. Ned. Akad. Wetensch., Afd. Natuurk., Sect. 2, 48(2): 21 (1952)

Virecta salicoïdes C.H.Wright, Bull. Misc. Inform. Kew 1898(1430): 302 (1898)

####### Type.

Cameroon, Mfoa, rocky bank of Mbei River, 00 Oct 1827, *G. L. Bates 527* (holotype: K; isotypes: BM, P).

####### Description.

Herb 25 cm high; stems flexible and tough; strongly rooted; leaves narrow 3–9 × 0.5–0.9 cm; inflorescence terminal.

####### Specimen examined.

Nkolebenga, northwest of d’Ebianemeyong, near Nyabesan, 60 km east of Campo, 2°25'N, 10°20'E, 11 Apr 1970. *R. Letouzey 10357* (YA).

####### Habitat.

Rocks at banks and beds of rivers and streams.

####### Distribution.

Cameroon (Fig. 22) and Gabon.

####### Conservation status in Cameroon.

*Virectariasalicoides* is not listed on http://www.iucnredlist.org, nor assessed by Onana and Cheek (2011). The taxon is currently known from two localities (Kribi and Ebianemeyong). The extent of occurrence of the species is estimated at less than 100 km^2^, and its area of occupancy is about 4 km^2^. The habitat is threatened by dam construction (Memve’ele Hydro-electric dam on Ntem waterfall at Nyabesan). Based on this threat, and the continuous decline of vegetation cover in the area, extent and /or quality of habitat *V.salicoides* is here assessed as Endangered. IUCN Red List Category: **Endangered EN B2ab (ii, iii).**

##### Sapindaceae

###### Deinbollia angustifolia

Taxon classificationPlantaeSapindalesSapindaceae

D.W.Thomas, Ann. Missouri Bot. Gard. 73(1): 219 (1986)

####### Type.

Cameroon – SouthWest Region, near Mundemba, 4°56'N, 8°52'E, rocky bank of Idu River at Bulu on path to Ekumbako, 10 m, 07 Mar 1984, *D. W. Thomas 3253* (holotype: MO; isotypes: K, P, YA).

####### Description.

Stenophyllous shrub of about 1 m high; roots spreading and deep; stems strong but flexible; leaves narrow 20–25 × 1–1.5 cm, grouped at the summit of the stem.

####### Specimens examined.

Korup Reserve, 4°55'N, 8°50'E, 16 Jul 1983, *D. W. Thomas 2243* (YA); between Bulu and Dibunda, 4°55'N, 8°52'E, 07 Mar 1984, *D. W. Thomas 3253* (YA); between Bulu and Ekumbako, 4°56'N, 8°52'E, 00 Jun 1984, *D. W. Thomas 3497* (YA).

####### Habitat.

Rocky bed of rivers, on banks of streams and rivers.

####### Distribution.

Cameroon (Fig. 23).

####### Conservation status in Cameroon.

*Deinbolliaangustifolia* is listed on http://www.iucnredlist.org as Vulnerable by Cheek (2017a). It was earlier assessed in Onana and Cheek (2011) as Vulnerable. The taxon is endemic to Cameroon and currently known from three localities. The extent of occurrence of this species is estimated less than 100 km^2^, and the area of occupancy is about 12 km^2^. The habitat of *D.angustifolia* is threatened by illegal timber logging. Based on this threat, and the continuous decline of vegetation cover in the area, extent and /or quality of habitat, the assessment of Cheek (2017a) is maintained as Vulnerable. IUCN Red List Category: **Vulnerable VU B2a b (ii, iii).**

###### Deinbollia saligna

Taxon classificationPlantaeSapindalesSapindaceae

Keay, Bull. Jard. Bot. État Bruxelles 26: 193 (1956)

####### Type.

Cameroon, Ndian, Kumba, 03 Mar 1936, *Smith Cam 80/36* (holotype: K, K000093228; isotype: FHI).

####### Description.

Shrub or small tree up to 2.5 m tall; sparingly to much branched; branches thick; stems strong and flexible; leaves clustered terminally; lanceolate 6–10 × 0.5–1 cm; flowers white.

####### Specimens examined.

Canyon of Ntem, 20 km southwest of Nyabessan, 01 Dec 1982, *B. A. Nkongmeneck 410* (YA); Mana River at Korup, 4°55'N, 8°50'E, 00 Dec 1983, *D. W. Thomas 2205* (YA); Ndian, on Ndian (Mana) waterfall, 4°56'N, 8°51'E, 17 Jan 1985, *D. W. Thomas 4268* (YA).

####### Habitat.

Inundated rocky bed and bank of rivers or streams.

####### Distribution.

Cameroon (Fig. 24), Ghana and Nigeria.

####### Conservation status in Cameroon.

*Deinbolliasaligna* is listed on www.iucnredlist.org. It was assessed globally as Vulnerable (World Conservation Monitoring Centre, 1998). In Onana and Cheek (2011) the species was assessed for the Red data Checklist of Cameroon as Vulnerable. This taxon is currently known from four localities. The extent of occurrence of the species is estimated at less than 100 km^2^, and the area of occupancy is about 16 km^2^. The localities are threatened by proposals for dam construction and timber extraction. Based on these threats, and the continuous decline of vegetation cover in the area, extent and /or quality of habitat *D.saligna* is here reassessed as Endangered: IUCN Red List Category: **Endangered ENB2ab (ii, iii).**

### Family in which all species are rheophytic (Podostemaceae)

The present study and herbarium data have shown that 43 species in 12 genera in the Podostemaceae (riverweed) family have so far been documented from the study area. The results revealed six monotypic genera: *Leiothylax*, *Letestuella*, *Stonesia*, *Tristicha*, *Winklerella*, and *Zehnderia*. The genus *Inversodicraea* has 10 species, *Saxicolella* has four while three genera *Dicraeanthus*, *Djinga*, and *Macropodiella* have two; and the largest genus in our area *Ledermanniella* has 17 species. In an earlier work Ameka et al. (2002) recognized 53 rheophytic species including 33 Podostemaceae for Cameroon. Since then 13 species have been added, 10 of which are in the Podostemaceae family. The Podostemaceae added within the last few years are the result of a deliberate search for the riverweed family in Cameroon. If similar efforts are made to collect the rheophytes in other African countries, many more species would be described for the continent. Apart from the Podostemaceae, the Hydrostachyaceae is the only other family in which all species are rheophytic in Africa (Ameka et al. 2002). So far the Hydrostachyaceae has not been recorded in the study area. Species of the two families are important since they are indicators of river health and also are the dominant macrophytes in tropical river systems; contributing to primary production and oxygenation of the river water (Quiroz et al. 1997, Ameka 2000). The Podostemaceae, for example, serve as substrate for epiphytic algae e.g., diatoms and cyanobacteria (blue-green algae) and other microscopic organisms (Ameka 2000). They also are habitats for invertebrate larvae (e.g., larvae of Simulium (black) fly, and nymphs of dragonfly and mayfly) that seek shelter and feed (Quiroz et al. 1997, Ameka per. obs.).

#### Podostemaceae

**Description.**Podostemaceae are annuals or perennials, that grow attached to rocks, in fast-flowing water, by rhizoids, or expanded holdfast; resembling algae or mosses. They produce flowers and fruits during the dry season when the water level in the rivers or streams drops.

##### 1. Dicraeanthus africanus

Taxon classificationPlantaeMalpighialesPodostemaceae

Engl., Bot. Jahrb. Syst. 38(1): 96 (1905)

Dicraeanthus ramosus H.E.Hess, Ber. Geobot. Inst. Eidg. Techn. Hochsch. Rübel Heft 32: 187 (1961).

###### Type.

Cameroon, *Winkler 901* (holotype: B).

###### Specimens examined.

Edea at Sanaga waterfall, *Annet 498* (P); 8 km S Kribi Lobe fall, *J.J. Bos 3590, 3887, 3888* (K, WAG); Ngaoundere, Vina waterfall, *Dulieu 4* (P); Natchigal, 62 km southeast of Bafia, *J. & A. Raynal 10544* (P).

###### Habitat.

River rapids and waterfalls.

###### Distribution.

Cameroon (Fig. 25), Gabon.

###### Conservation status in Cameroon.

*Dicraeanthusafricanus* has been assessed for the IUCN Red List, globally, in 2007 as Least Concern (Ghogue 2010b). This species was not assessed by Onana and Cheek (2011). Two localities, waterfalls, are known for this species. The extent of occurrence and the area of occupancy are both estimated at less than 10 km^2^. There are currently two major threats to the survival of this species at the Edea and Lobe waterfalls. First is the planned hydropower dam on the Sanaga River. Secondly the waterfalls on Lobe River is a big tourist attraction site. The dam across the river and activities (e.g., trampling) of tourists will adversely impact the habitats of the species and affect its survival. *D.africanus* is currently reassessed as Critically Endangered. IUCN Red List Category: **Critically Endangered CRB1+2ab (iii).**

##### 2. Dicraeanthus zehnderi

Taxon classificationPlantaeMalpighialesPodostemaceae

H.E.Hess, Ber. Geobot. Inst. Eidg. Techn. Hochsch. Stiftung Rŭbel Heft 32: 188 (1961)

###### Type.

Cameroon, Edea, 30 Jan 1951, *Zehnder 259* (syntype: Z, ZT).

###### Specimens examined.

Edea at Sanaga waterfall, *Zehnder 259, 260, 262* (ZT); Sanaga waterfalls, 30 Jan 1951, *Hess 51*/*270* (ZT).

###### Habitat.

Growing in river rapids and waterfalls of Sanaga.

###### Distribution.

Cameroon (Fig. 25).

###### Conservation status in Cameroon.

*Dicraeanthuszehnderi* was listed on http://www.iucnredlist.org as Critically Endangered (Ghogue 2010c). It was also assessed by Onana and Cheek (2011) as Critically Endangered since it has not been collected for many decades. The only known collecting locality for *D.zehnderi* is the waterfall on Sanaga River at Edea. The extent of occurrence and the area of occupancy are both estimated at 4 km^2^. The hydropower dam on the waterfall will adversely affect the survival of this species and other Podostemaceae species present at this locality. The taxon is here reassessed as Critically Endangered. IUCN Red List Category: **Critically Endangered CRB1+2ab (iii).**

##### 3. Djinga cheekii

Taxon classificationPlantaeMalpighialesPodostemaceae

Ghogue, Huber & Rutish., Nordic J. Bot. 31(4): 458 (2013)

###### Type.

Cameroon, near Manjo, 12 Jan 2011, *J. P. Ghogue 2125* (YA, Z, ZT).

###### Specimens examined.

Littoral Province, Mantem River, near Manjo, on the Douala – Nkongsamba highway, 4°49'N, 9°46'E, 12 Jan 2011, *J.-P. Ghogue 2126* and *2128* (K, YA, Z, ZT); Mbo River, Manjo (Manengole Village), 4°52'N, 9°51'E, 12 Dec 2004, *R. Imaichi, Y. Kita and J.-P*. *Ghogue CMR35* (TNS, Z, ZT).

###### Habitat.

River rapids.

###### Distribution.

Cameroon (Fig. 25).

###### Conservation status in Cameroon.

*Djingacheekii* is not listed on the http://www.iucnredlist.org. The taxon is known only from the type locality, Mantem River near Manjo. The extent of occurrence is estimated as 4 km^2^, and area of occupancy is about 4 km^2^. The main threat at the locality is agriculture. Based on this threat, and the continuous decline of vegetation cover in the area, extent and /or quality of habitat the taxon is here assessed as Critically Endangered. IUCN Red List Category: **Critically Endangered CRB1+2ab (iii).**

##### 4. Djinga felicis

Taxon classificationPlantaeMalpighialesPodostemaceae

C.Cusset, l. Cameroun 30: 58 (1987)

###### Type.

Cameroon, Adamawa, north of mount Djinga, 29 Oct 1967, *H. Jacques – Felix. 8889* (holotype: P).

###### Specimen examined.

Adamawa stream, north of mount Djinga, 29 Oct 1967, *Jacque-Félix 8889* (holo-P).

###### Habitat.

Mt. Djinga, Adamaoua, near Tignere, river rapids.

###### Distribution.

Cameroon (Fig. 25).

###### Conservation status in Cameroon.

This taxon, *D.felicis*, has not yet been assessed for the IUCN Red List. This species was assessed by Onana and Cheek (2011) as Critically Endangered since known from a single collection at the time. The species is most likely extinct at the type locality, Djinga Mts, Admmoua, north-western Cameroon. There are two other localities for the species, Juafef waterfall, where a hotel has been built, which is visited by many tourists coming into that area of Cameroon; and the other Anyajua waterfall is in an agricultural landscape, all in NW Cameroon (Ghogue et al. 2009). The extent of occurrence and area of occupancy are both estimated at 4 km^2^ each. Tourism and agricultural activities will adversely affect the habitat of the species. *D.felicis* is, therefore, reassessed currently as Critically Endangered. IUCN Red List Category: **Critically Endangered CRB1+2ab (iii).**

##### 5. Inversodicraea achoundongii

Taxon classificationPlantaeMalpighialesPodostemaceae

J.J.Schenk, R.Herschlag & D.W.Thomas, Syst. Bot. 40(2): 542 (2015)

###### Type.

Cameroon, West of Nyabezan, 01 Dec 1992, *D.W. Thomas & G. Achoundong 9642* (YA).

###### Specimen examined.

Ntem River, west of Nyabessan, 02°24'N, 10°22'E, 01 Dec 1992, *D. W. Thomas & G. Achoundong 9642* (YA).

###### Habitat.

Memve’ele waterfalls, Ntem River, alt. 395 m.

###### Distribution.

Cameroon (Fig. 25).

###### Conservation status in Cameroon.

*Inversodicraeaachoundongii* is yet to be assessed for the IUCN Red List. The taxon is currently known only from the type locality at Memve’ele waterfalls on the Ntem River. The extent of occurrence and area of occupancy are both estimated at 4 km^2^ each. The proposed hydropower dam on the Ntem River will certainly impact the survival of the species. Based on this threat, the species is here assessed as Critically Endangered. IUCN Red List Category: **Critically Endangered CRB1+2ab (iii).**

##### 6. Inversodicraea bosii

Taxon classificationPlantaeMalpighialesPodostemaceae

(C.Cusset) Rutish. & Thiv, Plant Syst. Evol. 283: 57 (2009)

Ledermanniella bosii C.Cusset, Bull. Mus. Natl. Hist. Nat., B, Adansonia 4: 385 (1984)

###### Type.

Cameroon, South of Kribi, 08 Jan 1969, *J.J. Bos 3592* (YA). Basionym: *Ledermanniellabosii* C.Cusset, Bull. Mus. Natl. Hist. Nat., B, Adansonia 4: 385 (1984).

###### Specimens examined.

South of Kribi on Lobe waterfall, 08 Jan 1969, *J.J. Boss 3592* (K, WAG, P); near Bongola, Ntem waterfall, Dec, *R. Letouzey 15333* (P); South Region, south Kribi, Lobe waterfall, 08 Jan 1969, *J.J. Boss 3597* (WAG).

###### Habitat.

Lobe waterfall, south of Kribi.

###### Distribution.

Cameroon (Fig. 25).

###### Conservation status in Cameroon.

*Inversodicraeabosii* is listed on http://www.iucnredlist.org. The taxon was assessed as Endangered (Ghogue 2017a). Onana and Cheek (2011) assessed this species earlier as Endangered. The species is known from two localities, Campo waterfalls and Lobe waterfalls at Kribi. The extent of occurrence and area of occupancy are both estimated at 4 km^2^ each. Based on the continuing decline in quality of the habitat of the species at Lobe waterfalls due to activities of tourists; and the possible adverse effect of the proposed hydropower dam near Campo waterfalls the assessment of Onana and Cheek (2011) and Ghogue (2017a) cannot be maintained. The species is here reassessed as Critically Endangered. IUCN Red List Category: **Critically Endangered CRBI+ B2ab (iii).**

##### 7. Inversodicraea cristata

Taxon classificationPlantaeMalpighialesPodostemaceae

Engl., Veg. Erde 9(3, 1): 274 (1915)

Ledermanniella cristata (Engl.) C.Cusset, Adansonia sér. 2, 14(2): 273 (1974)

###### Type.

Cameroon, near Malaka, Nov, *Ledermann 1173* (lectotype: B).

###### Specimens examined.

Near Malaka, 500 m alt., Nov, *Ledermann 1173, 1189* (U); Mari River waterfall, c. 8 km north of Betare Oya, 05 Feb 1966, *Leeuwenberg 7761* (WAG, YA); in Mvigili, northwest of Moan, 24 km southeast of Nyabezan, Mar, *J. & A. Raynal 10263* (P); Maan (24 km southeast of Nyabezan), rocky bank of Mvigili River, northwest of the village, 06 Mar 1963, *J. & A. Raynal 10263* (P, YA).

###### Habitat.

River rapids.

###### Distribution.

Angola, Cameroon (Fig. 25), Central African Republic, Equatorial Guinea, Gabon.

###### Conservation status in Cameroon.

*Inversodicraeacristata* is listed on http://www.iucnredlist.org. The taxon was assessed globally as Vulnerable (Ghogue 2017b). The species is known from five localities. The extent of occurrence of *I.critata* is about 73,144 km^2^ and the area of occupancy is about 24 km^2^. The main threats currently known from the localities are mining and agriculture. Based on these threats, and the continuous decline of vegetation cover in the area, extent and /or quality of habitat, *I.cristata* is currently assessed as Vulnerable IUCN Red List Category: **Vulnerable VUB1+2ab (iii).**

##### 8. Inversodicraea ebo

Taxon classificationPlantaeMalpighialesPodostemaceae

Cheek, Blumea 62: 125 (2017)

###### Type.

Cameroon, Yabassi, near Locndeng, 07 Dec 2013, *van der Burgt 1716* (YA).

###### Specimens examined.

Cameroon, Littoral Region, Yabassi, near Locndeng, Ebo River, 07 Dec 2013, *van der Burgt 1716* (YA).

###### Habitat.

On rocks in river rapids.

###### Distribution.

Cameroon (Fig. 25).

###### Conservation status in Cameroon.

*Inversodicraeaebo* is not listed on http://www.iucnredlist.org. The taxon is known only from the type locality. The extent of occurrence and the area of occupancy are both estimated at about 4 km^2^ each. The main threats at the locality are forest logging, mining and agriculture. The species is here assessed as Critically Endangered. IUCN Red List Category: **Critically Endangered CRB1+2ab (iii).**

##### 9. Inversodicraea eladii

Taxon classificationPlantaeMalpighialesPodostemaceae

Cheek, Blumea 62: 151 (2017)

###### Type.

Cameroon, Campo Ma`an area, 30 Nov 2001, *M. Elad & P. Tchouto 1485A* (YA).

###### Specimen examined.

Cameroon, South Region, Campo Ma’an area, Lobe, Lobe waterfalls, 30 Nov 2001, *M. Elad & P. Tchouto 1485A* (YA).

###### Habitat.

On rocks in waterfall near the sea, in evergreen forest zone.

###### Distribution.

Cameroon (Fig. 25).

###### Conservation status in Cameroon.

*Inversodicraeaeladii* as for the species before it is not as yet assessed for the IUCN Red List. The taxon is known from one locality. The extent of occurrence and the area of occupancy are both estimated at 4 km^2^ each. The main threat at the locality is touristic activity. The species is here assessed as Critically Endangered. IUCN Red List Category: **Critically Endangered CRB1+2ab (iii).**

##### 10. Inversodicraea kamerunensis

Taxon classificationPlantaeMalpighialesPodostemaceae

Engl., Veg. Erde 9(3, 1): 274 (1915)

Ledermanniella kamerunensis (Engl.) C.Cusset, Adansonia sér. 2, 14(2): 274 (1974).

###### Type.

Cameroon, Campo, near Dipikar, Aug, *Ledermann 440a* (YA).

###### Specimen examined.

Campo River waterfalls, near Dipikar, 00 Aug 1908, *Ledermann 440a* (YA).

###### Habitat.

Waterfalls in low altitudes.

###### Distribution.

Cameroon (Fig. 25).

###### Conservation status in Cameroon.

*Inversodicraeakamerunensis* is listed on http://www.iucnredlist.org as Vulnerable (Ghogue 2017c). It was assessed by Onana and Cheek (2011) as Critically Endangered. The species is endemic to Cameroon and known from only the type locality; the species has not been collected since 1908. The extent of occurrence and the area of occupancy are both estimated at 4 km^2^ each. The main threat at this locality is dam construction on Ntem River. *I.kamerunensis* is here reassessed and Critically Endangered status maintained. IUCN Red List Category: **Critically Endangered CRB1+2ab (ii, iii).**

##### 11. Inversodicraea ledermannii

Taxon classificationPlantaeMalpighialesPodostemaceae

Engl., Veg. Erde 9(3, 1): 274 (1915)

Ledermanniella ledermannii (Engl.) C.Cusset, Adansonia sér. 2, 14(2): 274 1974)

###### Type.

Cameroon, South Region, near Kribi, Grand Batanga, *Ledermann 225* (YA).

###### Specimens examined.

SW Region, Korup National Park, 5°01'N, 8°50'E, 50 m, 5–15 Dec 1984, *D.W. Thomas 4135A* (K, P); near Kribi, Lobe waterfalls, Grand Batanga, *Ledermann 225* (U); 6 km from Kribi, Lobe waterfalls, *De Wilde 2875* (P, YA).

###### Habitat.

River rapids.

###### Distribution.

Angola, Cameroon (Fig. 25), Côte d’Ivoire, Gabon, Guinea, Sierra Leone.

###### Conservation status in Cameroon.

*Inversodicraealedermannii* is listed on http://www.iucnredlist.org as Least Concern, globally (Diop 2017). The taxon is known from five localities. The extent of occurrence of *I.ledermannii* is about 29,454 km^2^ and the area of occupancy is about 20 km^2^. The main threats currently known from the localities are logging, agriculture and touristic activities. Based on these threats, and the continuous decline of vegetation cover in the area, extent and/or quality of habitat, *I.ledermannii* is currently reassessed as Vulnerable. IUCN Red List Category: **Vulnerable VUB2ab (iii).**

##### 12. Inversodicraea ntemensis

Taxon classificationPlantaeMalpighialesPodostemaceae

(Y.Kita, Koi, Rutish. & M.Kato) J.J.Schenk, R.Herschlag & D.W.Thomas, Syst. Bot. 40(2): 542. (2015)

Ledermanniella ntemensis Y.Kita, Koi, Rutish. & M.Kato; Acta Phytotax. Geobot. 59: 224 (2008)

###### Type.

Cameroon, *R. Imaichi Kita, Y. & J. P. Ghogue CMR 65* (YA). Basionym: *Ledermanniellantemensis* Y.Kita, Koi, Rutish. & M.Kato; Acta Phytotax. Geobot. 59: 224 (2008).

###### Specimens examined.

South Region, Canon of Ntem, 30 km southwest of Nyabessan, 01 Dec 1982, *Nkongmeneck 420* (YA); Ntem waterfalls, near Bongola, 40 km southeast of Campo, 10 Dec 1979, *R. Letouzey 15333* (P, YA); Campo a’an area, Memve’ele waterfalls, 2°24'N, 10°21'E, 17 Jan 2002, *P. Tchouto 3373* (K, KRI, SCA, WAG, YA).

###### Habitat.

Rapids of Ntem River.

###### Distribution.

Cameroon (Fig. 25).

###### Conservation status in Cameroon.

*Inversodicraeantemensis* is not listed on http://www.iucnredlist.org. It was assessed in Onana and Cheek (2011) as Critically Endangered. The taxon is endemic to Cameroon and to the Ntem River. The extent of occurrence is about 4 km^2^, and the area of occupancy is about 4 km^2^. The main threat to the survival of the species is dam construction on Ntem River. The species is here reassessed and maintained as Critically Endangered. IUCN Red List Category: **Critically Endangered CRB1+2ab (ii, iii).**

##### 13. Inversodicraea tchoutoi

Taxon classificationPlantaeMalpighialesPodostemaceae

Cheek, Blumea 62: 149 (2017)

###### Type.

Cameroon, *P. Tchouto 3378* (YA).

###### Specimens examined.

South Region, Campo Ma’an Area, Boucle du Ntem, near Meyas Ntem, 2°20'N, 10°35'E, 480 m alt., 16 Feb 2001, *P. Tchouto 3170* (K, KRI, SCA, WAG); Memve’ele waterfalls, 2°24'N, 10°21'E, 360 m alt., 17 Jan 2002, *P. Tchouto 3376* (K, KRI, SCA, *R. Letouzey 10299* (P).

###### Habitat.

Waterfalls in evergreen forest.

###### Distribution.

Cameroon (Fig. 25).

###### Conservation status in Cameroon.

*Inversodicraeatchoutoi* has not yet been assessed for the IUCN Red List. The taxon is known from only the Memve’ele waterfalls. The extent of occurrence is about 2 km^2^, and the area of occupancy is also about 2 km^2^. The main threat is the construction of a dam on the Ntem River and touristic activities. The species is here assessed as Critically Endangered. IUCN Red List Category: **Critically Endangered CRB1+2ab (ii, iii).**

##### 14. Inversodicraea xanderi

Taxon classificationPlantaeMalpighialesPodostemaceae

Cheek, Blumea 62: 147(2017)

###### Type.

Cameroon, Campo, 04 May 2016, *van der Burgt 1940* (holotype: K; isotypes: P, Z).

###### Specimens examined.

South Region, Campo, Campo-Ma’an National Park, north of the road Campo to Ma’an, 2°20'N, 10°13'E, 230 m alt., 04 Mar 2016, *van der Burgt 1940* (holotype: K; isotype: P, YA, Z).

###### Habitat.

On rocks in streams.

###### Distribution.

Cameroon (Fig. 25).

###### Conservation status in Cameroon.

*Inversodicraeaxanderi* is not listed on http://www.iucnredlist.org. The taxon is currently known only from Campo. The extent of occurrence is estimated at 4 km^2^, and the area of occupancy is also about 4 km^2^. No major threat is known from the locality where the species occurs, therefore, *I.xanderi* is currently assessed as Near Threatened. IUCN Red List Category: **Near Threatened (NT).**

##### 15. Ledermanniella aloides

Taxon classificationPlantaeMalpighialesPodostemaceae

(Engl.) C.Cusset, Adansonia sér. 2, 14(2): 273 (1974)

Inversodicraea aloides Engl., Veg. Erde 9(3, 1): 271 (1915)

###### Type.

Cameroon, Tschape pass, near Tchabal Mbabo, *Ledermann 2785* (lectotype: B; isotype: U).

###### Specimens examined.

Nigeria, Butum River, *Keay FHI 25150*; Utanga, Butum River, *Keay FHI 25153*; Tschape pass, near Tchabal Mbabo, *Ledermann 2785* (U).

###### Habitat.

On rocks in river.

###### Distribution.

Cameroon (Fig. 25) and Nigeria.

###### Conservation status in Cameroon.

*Ledermanniellaaloides* has been assessed globally as Vulnerable by Diop (2010a). Onana and Cheek (2011) assessed this taxon for Cameroon as Endangered. The taxon is known from one locality. The extent of occurrence, and the area of occupancy are both estimated at 4 km^2^ each. Based on the area of occupancy, the number of localities and the agricultural development impact in the area, *L.aloides* is here reassessed and maintained as Endangered. IUCN Red List Category: **Endangered EN B2ab (iii).**

##### 16. Ledermanniella batangensis

Taxon classificationPlantaeMalpighialesPodostemaceae

(Engl.) C.Cusset, Adansonia sér. 2, 14(2): 273 (1974)

Dicraeia batangensis Engl., Bot. Jahrb. Syst. 43(4): 380 (1909)
Inversodicraeia batangensis Engl., Veg. Erde 9(3, 1): 271 (1915)

###### Type.

Cameroon, Grand Batanga, *Ledermann 221* (holotype: B). Basionym: *Dicraeiabatangensis* Engl., Bot. Jahrb. Syst. 43(4): 380 (1909).

###### Specimen examined.

Grand Batanga, Lobe waterfalls, *Ledermann 221* (holotype: B, isotype: U).

###### Habitat.

On rocks in waterfalls.

###### Distribution.

Cameroon (Fig. 25).

###### Conservation status in Cameroon.

*Ledermanniellabatangensis* is listed on http://www.iucnredlist.org globally as Critically Endangered (Ghogue 2010d). Onana and Cheek (2011) also assessed this species as Critically Endangered. The taxon is known from only Lobe waterfalls. The area of occupancy and extent of occurrence are both estimated at 4 km^2^. The locality has a booming tourist industry and this has led to a general decline in quality of the habitat of the species. *L.batangensis* is here reassessed and the earlier assessment is maintained as Critically Endangered. IUCN Red List Category: **Critically Endangered B1ab (ii, iii) +2ab (ii, iii).**

##### 17. Ledermanniella bifurcata

Taxon classificationPlantaeMalpighialesPodostemaceae

(Engl.) C.Cusset, Adansonia sér. 2, 14(2): 273 (1974)

Inversodicraea bifurcata Engl., Veg. Erde 9(3, 1): 273 (1915)

###### Type.

Cameroon, Kribi, *Mildbraed 5951* (holotype: B).

###### Specimens examined.

Bipindi, *Annet 321* (P); 10 km from Kribi-Lolodorf, Kienke rapids, *J.J. Bos 7071, 7072* (WAG); 33 km northeast of Eta, 60 km southeast of Ngoila, Nki waterfalls, *R. Letouzey 11949* (P); 50 km east of Grand Batanga, Kribi waterfalls, *Mildbraed 5951, 5952, 5952a* (YA).

###### Habitat.

River rapids and waterfalls in evergreen forests.

###### Distribution.

Cameroon (Fig. 25), Gabon, Congo, Equatorial Guinea.

###### Conservation status in Cameroon.

*Ledermanniellabifurcata* has been assessed globally as Vulnerable (Ghogue 2010e). The taxon is known from 6 localities. The extent of occurrence of *L.bifurcata* is about 11,166 km^2^ and the area of occupancy is about 24 km^2^. The main threats currently known from the localities are forest logging and agriculture. Based on these threats, the number of localities currently known, and the continuous decline of vegetation cover in the area, extent and /or quality of habitat, *L.bifurcata* is currently reassessed as Vulnerable. IUCN Red List Category: **Vulnerable VUB1+2ab (iii).**

##### 18. Ledermanniella keayi

Taxon classificationPlantaeMalpighialesPodostemaceae

(G.Taylor) C.Cusset, Adansonia sér. 2, 14(2): 274 (1974)

Inversodicraea keayi G.Taylor; Bull. Brit. Mus. (Nat. Hist.) Bot. 1: 78 (1953)

###### Type.

Cameroon, Kumbo, *Keay FHI 28457* (holotype: K).

###### Specimens examined.

Cameroon: Banso, Bamenda, *Keay FHI 28457* (YA); near Sagbo, Ndop near Bamenda, 1800 m alt., *C. D. Adams 11073* (LISC); Kumbo, 1650 m alt., *Keay FHI 28457* (K).

###### Habitat.

River rapids.

###### Distribution.

Cameroon (Fig. 25).

###### Conservation status in Cameroon.

*Ledermanniellakeayi* is listed on http://www.iucnredlist.org as Critically Endangered (Diop 2010b). The taxon is known from one locality, restricted to a small area in an agricultural landscape. The extent of occurrence and the area of occupancy are both estimated at 2 km^2^ each. The earlier assessment is here maintained as Critically Endangered. IUCN Red List Category: **Critically Endangered CRB2ab (iii).**

##### 19. Ledermanniella letouzeyi

Taxon classificationPlantaeMalpighialesPodostemaceae

C.Cusset, Bull. Mus. Natl. Hist. Nat., B, Adansonia Sér. 4, 6(3): 260. (1985)

###### Type.

Cameroon, near Lokando, Mount Rumpi, Ure, 23 Mar 1976, *R. Letouzey 14517* (YA).

###### Specimen examined.

30 km northwest of Kumba, near Lokando, Mount Rumpi, Ure, on river, Mar, *R. Letouzey 14517* (holotype P, isotype YA).

###### Habitat.

River rapids and waterfalls in tropical forests.

###### Distribution.

Cameroon (Fig. 25).

###### Conservation status in Cameroon.

*Ledermanniellaletouzeyi* is listed on http://www.iucnredlist.org. The taxon was assessed as Endangered by Cheek (2004). Onana and Cheek (2011) maintained the Endangered status of Cheek (2004). The species is known from two localities. The extent of occurrence is estimated at 4 km^2^ and the area of occupancy is about 8 km^2^. The main threats in the locality are forest exploitation and agriculture. The earlier assessment by Cheek (2004) and Onana and Cheek (2011) is maintained. IUCN Red List Category: **Endangered ENB2ab (iii).**

##### 20. Ledermanniella linearifolia

Taxon classificationPlantaeMalpighialesPodostemaceae

Engl., Bot. Jahrb. Syst. 43(4): 378 (1909)

Sphaerothylax linearifolius Engl., Veg. Erde 9(3, 1): 275 (1915)

###### Type.

Cameroon, 28 Aug 1908, *C. Ledermann 440* (YA).

###### Specimens examined.

7 km south of Kribi, Lobe waterfall, Jan, *J. Bos3591* (K); 7 km south of Kribi, Lobe waterfalls, Aug, *De Wild 2876* (P, WAG, YA); Nkam, near Sahe, 3 km southwest Nkondjok road Bafang-Yabassi, Feb *R. Letouzey 11146* (P).

###### Habitat.

River rapids and waterfall.

###### Distribution.

Cameroon (Fig. 25).

###### Conservation status in Cameroon.

*Ledermanniellalinearifolia* is listed on http://www.iucnredlist.org. It was assessed as Endangered (Ghogue 2010f). Onana and Cheek (2011) reassessed this species and maintained the Endangered status. The taxon is known from 6 localities. The extent of occurrence of *L.linearifolia* is about 42,848,649 km^2^ and the area of occupancy is about 16 km^2^. The main threats currently known from the localities are agriculture and touristic activities. Based on these threats, the number of localities currently known, and the continuous decline of vegetation cover in the area, extent and /or quality of habitat, *L.linearifolia* is currently reassessed and maintained as Endangered. IUCN Red List Category: **Endangered ENB2ab (iii).**

##### 21. Ledermanniella monandra

Taxon classificationPlantaeMalpighialesPodostemaceae

(Engl.) C.Cusset, Adansonia, sér., 2, 14(2): 274 (1974)

Monandriella linearifolia Engl., Bot. Jahrb. Syst. 60(4): 457 (1926)

###### Type.

Cameroon, Mao Bika, near Dodeo, 05 Mar 1909, *C. Ledermann 2872* (YA).

###### Specimen examined.

Mao Bika, near Dodeo, 60 km west of Tignere, 700 m alt., Mar, *Ledermann 2872* (holotype B).

###### Habitat.

River rapids.

###### Distribution.

Cameroon (Fig. 25).

###### Conservation status in Cameroon.

*Ledermanniellamonandra* has not yet been assessed for the IUCN Red List, but it was assessed in Onana and Cheek (2011). The taxon is known from one locality. The extent of occurrence and the area of occupancy are estimated at 4 km^2^ each. Due to habitat degradation and the continuous decline of vegetation cover in the area, extent and /or quality of habitat, *L.monandra* is currently reassessed as Critically Endangered. IUCN Red List category: **Critically Endangered CRB2ab (iii).**

##### 22. Ledermanniella musciformis

Taxon classificationPlantaeMalpighialesPodostemaceae

(G.Taylor) C.Cusset, Adansonia ser 2 14(2): 274 (1974)

Inversodicraea musciformis G.Taylor; Bull. Brit. Mus. (Nat. Hist.) Bot. 1: 75 (1953)

###### Type.

Cameroon, Mba Kokeka, near Bamenda, Jan, *Keay FHI 28542* (holotype: K). Basionym: *Inversodicraeamusciformis* G.Taylor; Bull. Brit. Mus. (Nat. Hist.) Bot. 1: 75 (1953).

###### Specimens examined.

Northwest slopes of Mts. Mba Kokeka, near Bamenda, Jan, *Keay FHI 28542* (K); Tchamba, Nakalba, 21 km southwest of Tchamba 1200 m alt., Jan, *J. & A. Raynal 13166* (P).

###### Habitat.

River rapids and waterfalls.

###### Distribution.

Cameroon (Fig. 25).

###### Conservation status in Cameroon.

*Ledermanniellamusciformis* is listed on http://www.iucnredlist.org as Data Deficient (Diop 2010c). Onana and Cheek (2011) reassessed this species as Endangered. This taxon is endemic to Cameroon and known from at least four localities. The extent of occurrence of *L.musciformis* is about 68,419,636 km^2^ and area of occupancy is about 16 km^2^. The main threat currently known from the localities is deforestation and agriculture. Based on these threats, the number of localities, and the continuous decline of vegetation cover in the area, extent and/or quality of habitat, *L.musciformis* is currently reassessed and Endangered status maintained. IUCN Red List Category: **Endangered ENB2ab (iii).**

##### 23. Ledermanniella onanae

Taxon classificationPlantaeMalpighialesPodostemaceae

Cheek, Kew Bull. 58: 733 (2003)

###### Type.

Cameroon, Bakossi Mts, northwest of Muambong, 04 Feb 1998, *J.-M. Onana 558* (K, YA).

###### Specimens examined.

Cameroon: South West Province, Bakossi Mts., Chide River falls, northwest of Muambong, 04 Feb 1998, *J.-M. Onana 558* (YA, K); South West Province, Bakossi Mts., Ndip River rapids between Nzimbeng and Kodmin, alt. 1150 m. fl. & fr., 14 Feb 1998, *M. Cheek 9196* (K, YA).

###### Habitat.

Perennial waterfalls and river rapids in submontane forest.

###### Distribution.

Cameroon (Fig. 25) and Gabon.

###### Conservation status in Cameroon.

*Ledermanniellaonanae* is listed on http://www.iucnredlist.org as globally Endangered (Ghogue 2010g). Onana and Cheek (2011) maintained the Endangered status. The taxon is known from three localities, two of which are on the same river. The extent of occurrence of *L.onanae* is about 23,751 km^2^ and the area of occupancy is about 12 km^2^. The main threats currently known from the localities are forest logging and agriculture. Based on these threats, and the continuous decline of vegetation cover in the area, extent and /or quality of habitat, *L.onanae* is currently reassessed and the Endangered status maintained. IUCN Red List Category: **Endangered ENB2ab (iii).**

##### 24. Ledermanniella pollardiana

Taxon classificationPlantaeMalpighialesPodostemaceae

Cheek & Ameka, Nordic J. Bot. 26: 214 (2008)

###### Type.

Cameroon, North Western Province, Bali, 1 km east, 5°52'N, 10°01'E, 19 Nov 2000, *B. Pollard 536* (K, YA).

###### Specimens examined.

Cameroon, North-western Province, Bali, 1 km east, 5°52'N, 10°01'E, 1280 m alt. fl., 19 Nov 2000, *B. Pollard 536* (K, YA).

###### Habitat.

Perennial waterfall, in full sun, in deforested area.

###### Distribution.

Cameroon (Fig. 25).

###### Conservation status in Cameroon.

*Ledermanniellapollardiana* is not as yet assessed for the IUCN Red List. Onana and Cheek (2011) assessed this species as Critically Endangered. The taxon is endemic to Cameroon, and known from only the type locality. The extent of occurrence and the area of occupancy are both estimated at 4 km^2^ each. Agricultural activities in the general area of the locality; with increased turbidity and siltation from agricultural practices will adversely affect the species. The species is here reassessed and Critically Endangered status maintained. IUCN Red List Category: **Critically Endangered CRB2ab (iii).**

##### 25. Ledermanniella prasina

Taxon classificationPlantaeMalpighialesPodostemaceae

J.J.Schenk & D.W.Thomas, Novon 14(2): 227 (2004)

###### Type.

Cameroon, 01 Dec 1990, *D.W. Thomas 11550* (K, WAG, YA).

###### Specimen examined.

Cameroon, 01 Dec 1990, *D.W. Thomas 11550*, [K, WAG, YA].

###### Habitat.

River rapids.

###### Distribution.

Cameroon (Fig. 25).

###### Conservation status in Cameroon.

*Ledermanniellaprasina* was assessed as Vulnerable (Cheek 2017b) for the IUCN Red List. The taxon is known only from the Mana River valley system in Cameroon. The extent of occurrence and the area of occupancy are both estimated at 2 km^2^ each. The assessment of Cheek (2017b) is maintained since no major changes have taken place at the locality since that assessment. The species is here reassessed as Vulnerable. IUCN Red List Category: **Vulnerable VUB1+2ab (iii).**

##### 26. Ledermanniella pusilla

Taxon classificationPlantaeMalpighialesPodostemaceae

(Warm.) C.Cusset, Adansonia ser. 2, 14(2): 274 (1974)

Dicraeanthus pusillus C.H.Wright, Fl. Trop. Afr. 6(1.1): 127 (1909)
Sphaerothylax pusilla Warm Kongel. Danske Vidensk. Selsk. Skr., Naturvidensk. Math. Afd. VI, 9: 146 (1899)

###### Type.

Cameroun, Bipindi, *G. Zenker 1050* (holotype: B; isotype: G). Basionym: *Sphaerothylaxpusilla* Warm., Kongel. Danske Vidensk. Selsk. Skr., Naturvidensk. Math. Afd. VI, 9: 146 (1899).

###### Specimens examined.

7 km south of Kribi, Lobe waterfall, *J.J. Bos 3598* (WAG); Lokoundje waterfall, Bipindi, *G. Zenker 1050* (G, K, L, M, U, Z).

###### Habitat.

Waterfalls.

###### Distribution.

Cameroon (Fig. 25), Democratic Republic of Cong and Gabon.

###### Conservation status in Cameroon.

*Ledermanniellapusilla* is listed on http://www.iucnredlist.org globally as Endangered (Ghogue 2010h). The taxon is known from two localities. The extent of occurrence is about 9,042 km^2^ and the area of occupancy is about 16 km^2^. The waterfalls at Lobe are a huge tourist center and the activities have caused a deterioration in the habitat of the species. The species is here reassessed as Endangered. IUCN Red List Category: **Endangered ENB1 + 2ab (iii).**

##### 27. Ledermanniella sanagaensis

Taxon classificationPlantaeMalpighialesPodostemaceae

C.Cusset, Bull. Mus. Natl. Hist. Nat. B, Adansonia Sér. 4, 6(3): 256 (1985)

###### Type.

Cameroon, Natchigal, *J. & A. Raynal 10543* (YA).

###### Specimens examined.

Cameroon, *J. & A. Raynal 10543* (YA); Natchigal, Sanaga waterfall, *A. & J. Raynal 10542* (P).

###### Habitat.

River rapids and waterfalls.

###### Distribution.

Cameroon (Fig. 25).

###### Conservation status in Cameroon.

*Ledermanniellasanagaensis* is listed on http://www.iucnredlist.org as Critically Endangered (Ghogue 2010i). The taxon is endemic to Cameroon, and known only from the Sanaga waterfall at Natchigal. The extent of occurrence and area of occupancy are both estimated at 4 km^2^ each. There is a proposal to build a dam at the locality of the species. Based on this threat the earlier assessment of Ghogue (2010i) as Critically Endangered is maintained. IUCN Red List Category: **Critically Endangered B2ab (ii, iii).**

##### 28. Ledermanniella schlechteri

Taxon classificationPlantaeMalpighialesPodostemaceae

(Engl.) C.Cusset, Adansonia sér., 2, 14(2): 275 (1974)

Dicraeia schlechteri Engl., Bot. Jahrb. Syst. 43: 381 (1909)
Inversodicraeia tenuissima Hauman, Bull. Jard. Bot. État 17: 180 (1944)

###### Type.

Congo Democratic Republic, 01 Jun 1899, *R. Schlechter12574* (K). Basionym: *Dicraeiaschlechteri* Engl., Bot. Jahrb. Syst. 43: 381 (1909).

###### Specimens examined.

Dehane, between Edea and Kribi, Jun, *Annet 459* (P).

###### Habitat.

River rapids and waterfalls.

###### Distribution.

Cameroon (Fig. 25), Congo and Democratic Republic of Congo.

###### Conservation status in Cameroon.

*Ledermanniellaschlechteri* is listed on http://www.iucnredlist.org as Vulnerable, globally (Ghogue 2010j). The taxon is known from two localities. The extent of occurrence is less than 100 km^2^, and the area of occupancy is about 8 km^2^. The proposed dam at Edea waterfall will further deteriorate the quality of the habitat of the species at that locality. Base on this threat and the number of localities where the species is currently found, *L.schlechteri* is here reassessed as Endangered. IUCN Red List Category: **Endangered ENB1+2ab (iii).**

##### 29. Ledermanniella thalloidea

Taxon classificationPlantaeMalpighialesPodostemaceae

(Engl.) C.Cusset, Adansonia sér 2, 14(2): 275 (1974)

Inversodicraeia thalloidea Engl., Veg. Erde 9(3, 1): 274 (1915)

###### Type.

Cameroon, Ndoungue near Nkongsamba, *Ledermann 6328a* (lectotype: B; isotypes: BM, U). Basionym: *Inversodicraeiathalloidea* Engl., Veg. Erde 9(3, 1): 274 (1915).

###### Specimens examined.

Tributary of Sanaga, 10 km north of Edea, *Kers 1904* (LISC); Ndoungue near Nkongsamba, 800 m alt., *Ledermann 6328a* (BM, U); Natchigal, Sanaga waterfall, *A. & J. Raynal 10542* (P).

**Habitat**. River rapids and waterfalls in tropical rain forest.

###### Distribution.

Cameroon (Fig. 25).

###### Conservation status in Cameroon.

*Ledermanniellathalloidea* is listed on http://www.iucnredlist.org as Endangered (Ghogue 2010k). However, Onana and Cheek (2011) assessed the taxon as Vulnerable. The taxon is endemic to Cameroon and known from two localities. The species’ area of occupancy is estimated to be less than 10 km^2^, and the extent of occurrence estimated at 18 km^2^. There is a decline in the quality of the habitat of the species; there is a dam at the Sanaga waterfalls, and another dam construction is in progress at the Nachtigal waterfalls, the two sites for the species. Due to the impact of the dams on the habitat of the species it is here reassessed as Critically Endangered. IUCN Red List Category: **Critically Endangered CRB2ab (iii).**

##### 30. Ledermanniella raynaliorum

Taxon classificationPlantaeMalpighialesPodostemaceae

C.Cusset, Bull. Mus. Natl. Hist. Nat. B, Adansonia Ser. 4, 6(3): 264 (1985)

###### Type.

Cameroon, 14 Jan 1965, *J. & A. Rayanal 12988* (YA).

###### Specimen examined.

Cameroon, 14 Jan 1965, *J. & A. Rayanal 12988* (YA).

###### Habitat.

River rapids.

###### Distribution.

Cameroon (Fig. 25).

###### Conservation status in Cameroon.

*Ledermanniellaraynaliorum* is not listed on http://www.iucnredlist.org. Onana and Cheek (2011) assessed this species as Endangered. The taxon is known from only one locality. The extent of occurrence and the area of occupancy are estimated at about 4 km^2^ each. Forest degradation is the main threat at the locality. The species is here assessed as Near Threatened. IUCN Red List Category: **Near Threatened (NT).**

##### 31. Ledermanniella variabilis

Taxon classificationPlantaeMalpighialesPodostemaceae

(G.Taylor) C.Cusset, Adansonia sér 2, 14(2): 275 (1974)

Inversodicraeia variabilis G.Taylor, Bull. Brit. Mus. (Nat. Hist.) Bot. 1: 75 (1953)

###### Type.

Cameroon, Manfe, Munaya, *Keay FHI 28688*, (holotype: K). Basionym: *Inversodicraeiavariabilis* G.Taylor, Bull. Brit. Mus. (Nat. Hist.) Bot. 1: 75 (1953).

###### Specimens examined.

Lobe waterfall, 7 km south of Kribi, *J.J. Boss 3594* (WAG); Mamfe, Munaya River, *Keay FHI 28688* (K).

###### Habitat.

River rapids and waterfalls.

###### Distribution.

Cameroon (Fig. 25).

###### Conservation status in Cameroon.

*Ledermanniellavariabilis* is listed on http://www.iucnredlist.org as Endangered (Ghogue 2010l). Onana and Cheek (2011) reassessed this species as Endangered. The species is known from two localities and the area of occupancy and extent of occurrence are estimated to be less than 10 km^2^ each. The Lobe waterfall locality is a famous tourist attraction so there is a continuous decline in the quality of the habitat at this site. The species is here reassessed as Critically Endangered. IUCN Red List Category: **Critically Endangered CRB1+2ab (ii, iii).**

##### 32. Leiothylax quangensis

Taxon classificationPlantaeMalpighialesPodostemaceae

Warm., Danske Vid. Selsk. Skrift. Ser.. VI, 9: 147 (1899)

Dicraeia quangensis Engl., Bot. Jahrb. Syst. 20(1–2): 134 (1894)
Leiocarpodicraea buesgenii Engl., Engl. Bot. Jahrb. Syst. 60: 465 (1926)
Leiothylax buesgenii Warm. ex Engl., Nat. Pflanzenfam. 18 a ed. 2, 58 (1930)
Leiothylax edeensis Engl. Nat. Pflanzenfam. 18 a ed. 2, 58 (1930)

###### Type.

Democratic Republic of Congo, *Teuscz in von Mechow’s Expedition 506* (holotype: M; isotype: G).

###### Specimens examined.

Edea, Sanaga waterfall, *Buesgen 439* (M); Sanaga waterfall, *Buesgen s.n.* (B, U).

###### Habitat.

River rapids and waterfalls in tropical rain forests.

###### Distribution.

Angola, Cameroon (Fig. 25), Democratic Republic of Congo.

###### Conservation status in Cameroon.

*Leiothylaxquangensis* has been assessed for the IUCN Red List as Endangered (Ghogue 2010m). The taxon is known from one locality, Edea waterfalls. The area of occupancy and extent of occurrence are both estimated at 2 km^2^ each. The hydropower dam at Edea will certainly impact the quality of the habitat of the species. *L.quangensis* is here reassessed as Critically Endangered. IUCN Red List Category: **Critically Endangered CRB1+2ab (ii, iii).**

##### 33. Letestuella tisserantii

Taxon classificationPlantaeMalpighialesPodostemaceae

G.Taylor, Bull. Brit. Mus. (Nat. Hist.) Bot. 1: 57 (1953)

Letestuella chevalieri G.Taylor, Bull. Brit. Mus. (Nat. Hist.), Bot. 1: 59 (1953)
Leiothylax warmingii (Engl.) Warm., Danske Vid. Selsk. Skrift. Ser. VI. ix 150 (1899)

###### Type.

Central African Republic, *Tisserant 1769* (holotype: BM; isotype: P).

###### Specimens examined.

Near Goyoum in Sanaga River, 20 km west of Deng Deng, *F.J. Breteler 981* (WAG); Plateau de l’Adamaoua, Vina waterfall, 15 km from Ngaoundere, *Zehnder 163* (ZT).

###### Habitat.

River rapids and waterfalls in tropical rain forest.

###### Distribution.

Benin, Cameroon (Fig. 25), Central African Republic, Mali, Namibia and Niger.

###### Conservation status in Cameroon.

*Letestuellatisserantii* is listed on http://www.iucnredlist.org as Least Concern (Diop 2010d) since it occurs in many countries. The taxon is known from two localities in Cameroon. The extent of occurrence is less than 100 km^2^, and the area of occupancy is less than 10 km^2^. Dams on the Sanaga River and agricultural activities are the main threats to the species. Based on the threats and the number of localities where the species is found, *L.tisserantii* is here assessed as Endangered. IUNC Red List Category: **Endangered ENB2ab (iii).**

##### 34. Macropodiella heteromorpha

Taxon classificationPlantaeMalpighialesPodostemaceae

(Baill.) C.Cusset, Adansonia, sér. 2, 17(3): 298 (1978)

Sphaerothylax heteromorpha Baill., Bull. Mens. Soc. Linn. Paris ii 876 (1890)
Macropodiella mildbraedii Engl., Bot. Jahrb. Syst. 60(5): 466 (1926)

###### Type.

Gabon, *Thollon 729* (holotype: P). Basionym: *Sphaerothylaxheteromorpha* Baill., Bull. Mens. Soc. Linn. Paris ii 876 (1890).

###### Specimens examined.

Makak Forest Reserve, *P. Bamps 1453* (YA); Nyong River, near Mbalmayo, *Mildbraed 7749, 7750* (B, U).

###### Habitat.

River rapids and waterfalls in tropical rainforests.

###### Distribution.

Cameroon (Fig. 25), Côte d’Ivoire and Gabon.

###### Conservation status in Cameroon.

*Macropodiellaheteromorpha* is listed on http://www.iucnredlist.org as Vulnerable (Ghogue 2010n). The taxon is known from four localities, and the area of occupancy is less than 500 km^2^ and the extent of occurrence estimated at 75 km^2^. At one of the localities (Meve’ele waterfalls) it is proposed to build a hydropower dam. The species is threatened by habitat decline due to future dam construction. According to Ghogue (2010n) two other localities, Nyong River near Mbalmayo and the Mpoume waterfalls on the Nyong River near Makak, have been listed by Cusset (1987) for this species. These two sites have been surveyed by Ghogue but the species has not been seen or collected. *M.heteromorpha* is, therefore, here reassessed as Critically Endangered. IUCN Red List Category: **Critically Endangered CRB2ab (ii, iii).**

##### 35. Macropodiella pellucida

Taxon classificationPlantaeMalpighialesPodostemaceae

(Engl.) C.Cusset, Fl. Cameroun 30: 64 (1987)

###### Type.

Cameroon, Bare, near Nkongsamba, *Ledermann 6142* (lectotype: BM). Basionym: *Inversodicraeapellucida* Engl., Veg. Erde 9(3, 1): 271, 272. (1915).

###### Specimens examined.

Bare, near Nkongsamba, on rocks in a waterfall, *Ledermann 6142* (BM); Ndian River, near Mundemba, Dec, *D.W. Thomas 2552* (MO, P).

###### Habitat.

River rapids and waterfalls in rainforest.

###### Distribution.

Cameroon (Fig. 25).

###### Conservation status in Cameroon.

*Macropodiellapellucida* is listed on http://www.iucnredlist.org as Endangered (Ghogue 2010o). Onana and Cheek (2011) maintained the Endangered status of Ghogue (2010o). The taxon is endemic to Cameroon and known from two localities. The extent of occurrence is less than 4 km^2^ and the area of occupancy of this species is estimated at less than 20 km^2^. There have not been further threats at the habitat of the species since the previous assessment. The species is reassessed as Endangered, maintaining the previous status. IUCN Red List Category: **Endangered ENB2ab (iii).**

##### 36. Saxicolella flabellata

Taxon classificationPlantaeMalpighialesPodostemaceae

(G.Taylor) C.Cusset, Fl. Cameroun 30: 94 (1987)

Pohliella flabellata G.Taylor, Bull. Brit. Mus. (Nat. Hist.), Bot. 1: 53 (1953)

###### Type.

Nigeria, Afi River, Dec, *Keay FHI 28240* (K). Basionym: *Pohliellaflabellata* G.Taylor, Bull. Brit. Mus. (Nat. Hist.), Bot. 1: 53 (1953).

###### Specimens examined.

Nigeria: Afi River, on Aboabam-Boje path, Dec, *Keay FHI 28240* (K); Cameroon: Ndian, near Mundemba, *D.W. Thomas 2654* (MO, P).

###### Habitat.

Submerged on rocks in fast-flowing river.

###### Distribution.

Cameroon (Fig. 25) and Nigeria.

###### Conservation status in Cameroon.

*Saxicolellaflabellata* is list on http://www.iucnredlist.org. The taxon has been assessed as Data Deficient (Ouedraogo 2010a) since species distribution, population status and threats to the species are unknown at the time of the assessment. Onana and Cheek (2011) reassessed the species as Endangered. The species is found in two localities. The extent of occurrence is estimated at 2 km^2^ and the area of occupancy is about 8 km^2^. The main threats at the localities are described as forest exploitation and agriculture. The species is here reassessed as Endangered. IUCN Red List Category: **Endangered ENB2ab (iii).**

##### 37. Saxicolella laciniata

Taxon classificationPlantaeMalpighialesPodostemaceae

(Engl.) C.Cusset, Fl. Cameroun 30: 94 (1987)

Inversodicraea laciniata Engl., Veg. Erde 9(3, 1): 271 (1915)
Pohliella laciniata Engl., Bot. Jahrb. Syst. 60(4): 458 (1926)

###### Type.

Cameroon, near Babong, *Ledermann 1185* (holotype: B). Basionym: *Pohliellalaciniata* Engl., Bot. Jahrb. Syst. 60(4): 458 (1926).

###### Specimens examined.

Dinger River near Babong, *Ledermann 1185* (B); Bawan River, on path to Agborkem (ex Ossidinge) at Tabo, 20 km west of Mamfe, *R. Letouzey 13731* (YA).

###### Habitat.

River rapids.

###### Distribution.

Cameroon (Fig. 25).

###### Conservation status in Cameroon.

*Saxicolellalaciniata* is listed on http://www.iucnredlist.org as Vulnerable (Ghogue 2010p). It was assessed in Onana and Cheek (2011) as Endangered. The taxon is known from two localities. The extent of occurrence is estimated at 2 km^2^ and the area of occupancy is 8 km^2^. The main threats at the localities are forest exploitation for agriculture purposes. The species is here reassessed and the Endangered status maintained. IUCN Red List Category: **Endangered ENB2ab (iii).**

##### 38. Saxicolella marginalis

Taxon classificationPlantaeMalpighialesPodostemaceae

(G.Taylor) C.Cusset ex Cheek, Pl Mount Oku & Ijim Ridge, Cameroon, Conserv. Checklist 153 (2000)

Butumia marginalis G.Taylor, Bull. Brit. Mus. (Nat. Hist.) Bot. 1: 55 (1953).

###### Type.

Nigeria, 25 Dec 1948, *Keay, Savory & Russell, #25152* (BM). Basionym: *Butumiamarginalis* G.Taylor, Bull. Brit. Mus. (Nat. Hist.) Bot. 1: 55 (1953).

###### Specimens examined.

Nigeria: Butum River, Utanga, 2 miles north of Bagga, Obudu, 25 Dec 1948, *Keay, Savory & Russell FHI 25152* (YA); Cameroon: Fundong, 22 Nov 1996, *M. Cheek 8740* (YA).

###### Habitat.

On smooth granite rocks in swift-flowing stream or river.

###### Distribution.

Cameroon (Fig. 25) and Nigeria.

###### Conservation status in Cameroon.

*Saxicolellamarginalis* is listed on http://www.iucnredlist.org. The taxon has been assessed globally as Critically Endangered (Ouedraogo 2010b). There is only one known collecting locality in the country. According to Ouedraogo (2010b) there is decline in the quality of the habitat due to pollution from laundry operations in the town of Fundong upstream of the site of this species. Based on this threat the assessment of Critically Endangered is maintained. IUCN Red List Category: **Critically Endangered B1ab (iii) +2ab (iii)**.

##### 39. Saxicolella nana

Taxon classificationPlantaeMalpighialesPodostemaceae

Engl., Bot. Jahrb. Syst. 60(4): 456 (1926)

###### Type.

Cameroon, near Mbalmayo, *Mildbraed 7749a* (holotype: B; isotype: U).

###### Specimens examined.

Cameroon, near Mbalmayo, Nyong River, 644 m alt, *Mildbraed 7749a* (YA); 11°27'N, 3°22'E, 28 Feb 2007, *M. Kato, R. Imaichi, S. Koi, & N. Katayama CMR-129* (YA).

###### Habitat.

River rapids and waterfalls.

###### Distribution.

Cameroon (Fig. 25).

###### Conservation status in Cameroon.

*Saxicolellanana* is listed on http://www.iucnredlist.org as Vulnerable (Ghogue 2010q). Onana and Cheek (2011) assessed this species as Critically Endangered, since at that time it had not been collected since the first collection many years ago. The taxon is endemic to Cameroon and is known from only Nyong River near Mbalmayo. The species was, however, collected again by Kato and associates in 2007. The area of occupancy of this species can be estimated to be less than 20 km^2^. The extent of occurrence -estimated to be less than 2 km^2^. The main threat is agricultural activity. The species is here reassessed as Endangered. IUCN Red List Category: **Endangered ENB2ab (ii, iii).**

##### 40. Stonesia ghoguei

Taxon classificationPlantaeMalpighialesPodostemaceae

E.Pfeiter & Rutish., Novon 19(1): 103 (2009)

###### Type.

Cameroon, Adamawa, Ngaoundere, 16 Feb 2005, *J. P. Ghogue 1665* (YA, K, Z).

###### Specimens examined.

Adamawa, Ngaoumdere, Tello Waterfalls, 16 Feb 2005, *J. P. Ghogue 1665* (YA, K, Z).

###### Habitat.

Growing in waterfalls, Tello Waterfalls, Ngaoundéré, Adamawa (Cameroon).

###### Distribution.

Cameroon (Fig. 25).

###### Conservation status in Cameroon.

*Stonesiaghoguei* is listed on http://www.iucnredlist.org as Vulnerable (Cheek 2018). Pfeifer et al. (2009) assessed *S.ghoguei* as Vulnerable. The taxon is endemic to Cameroon and known from only one collecting locality, Tello Waterfalls. The extent of occurrence and area of occupancy are estimated at 4 km^2^ each. Their assessment is retained for now. IUCN List Category: **Vulnerable VU D1 + 2**.

##### 41. Tristicha trifaria

Taxon classificationPlantaeMalpighialesPodostemaceae

(Bory ex Willd.) Spreng., Systema Vegetabilium 1 (1824)

Dufourea boryi A.Rich., Dict. Class. Hist. Nat. 5: 636 (1824)
Dufourea hypnoides St-Hil. Mém. Mus. Hist. Nat. 10: 472 (1823)
Dufourea trifaria Bory ex Willd., Sp. Pl., ed. 4 5(1): 55 (1810)
Dufourea alternifolia Willd., Mag. Neuesten Entdeck. Gesammten Naturk. Ges. Naturf. Freunde Berlin 6: 64 (1812)
Tristicha alternifolia Thouars ex Spreng., Syst. Veg. ed. 16(1): 22 (1824)
Tristicha alternifolia Thouars, ex Roem. & Schult. Syst. Veg. i. 50 (1817)

###### Type.

Mauritius, *Bory de St Vincent s.n*. (holotype: B; isotype: P).

###### Specimens examined.

8 km south of Kribi, Lobe waterfall, *J.J. Bos 3593* (YA); Vina waterfall, near Ngaoundere, Feb, *Dulieu 5* (ALF); Hossere Koum, 40 km west of Tchollire, Nov, *Fotius 2418* (P); Limbe (Joke River), Mar, *Brenen 9495, 9496* (BR, COL, P, SRGH); 8 km North Betare Oya (Mari River fall), Nov, *Leeuwenberg 7767* (P, WAG); Roua 20 km northeast of Mokolo, Oct, *R. Letouzey 7280* (P); Sahe, 3 km southwest of Nkondjok (Bafang – Yabassi Road), Nkam River, Feb, *R. Letouzey 11145* (P).

###### Habitat.

River rapids and waterfalls.

###### Distribution.

AFRICA; Angola, Benin, Burkina Faso, Cameroon (Fig. 25), Cote d’Ivoire, Democratic Republic of Congo, Ethiopia, Ghana, Guinea, Kenya, Liberia, Madagascar, Malawi, Mali, Mauritius, Mascarene Islands, Mozambique, Namibia, Niger, Nigeria, Senegal, Sierra Leone, South Africa, Sudan, Tanzania, Togo, Uganda, Zambia, Zimbabwe. CENTRAL AND SOUTH AMERICA; Argentina, Brazil, Columbia, Costa Rica, Cuba, El Salvador, Guatemala, Mexico, Nicaragua, Panama, Paraguay, Uruguay, Venezuela.

###### Conservation status in Cameroon.

*Tristichatrifaria* is not listed on www.iucnredlist.org. The extent of occurrence of *T.trifaria* is about 12,000 km^2^ and the area of occupancy is about 28 km^2^. The taxon is currently known from about 7 localities. Lobe area, one of the localities where the species is found is a famous touristic site. Also agricultural activities are on the increase in other localities. Based on these threats, the number of localities and the continuous decline of vegetation cover in the area, extent and /or quality of the habitat, *T.trifaria* is here assessed as Vulnerable. IUCN Red List Category: **Vulnerable VUB1 + 2ab (ii, iii).**

##### 42. Winklerella dichotoma

Taxon classificationPlantaeMalpighialesPodostemaceae

Engl., Bot. Jahrb. Syst. 38(1): 97 (1905)

###### Type.

Cameroon, Edea, 30 Jan 1951, *Winkler 900* (holotype: B).

###### Specimens examined.

Edea waterfall, *Winkler 900* (B); Edea waterfall, 30 Jan 1951, *Zehnder 271, 275, 277* (BR, ZT).

###### Habitat.

River rapids and waterfalls.

###### Distribution.

Cameroon (Fig. 25).

###### Conservation status in Cameroon.

*Winklerelladichotoma* is list on http://www.iucnredlist.org as Critically Endangered (Ghogue 2010r). The species is endemic to Cameroon and only known from the Edea waterfalls on the Sanaga River. There is a hydropower dam built on the river at the collecting locality of the species. The area of occupancy and extent of occurrence are estimated at 2 km^2^ each. The earlier assessment of Ghogue (2010r) as Critically Endangered is maintained. IUCN Red List Category: **Critically Endangered B1ab (iii) +2ab (iii).**

##### 43. Zehnderia microgyna

Taxon classificationPlantaeMalpighialesPodostemaceae

C.Cusset, Fl. Cameroon 30: 56 (1987)

###### Type.

Cameroon, Edea, 29 Jan 1951, *Zehnder 264* (holotype: ZT).

###### Specimen examined.

Edea, Sanaga waterfall, 29 Jan 1951, *Zehnder 264, 276, 278* (ZT).

###### Distribution.

Cameroon (Fig. 25).

###### Conservation status in Cameroon.

*Zehnderiamicrogyna* is listed on http://www.iucnredlist.org. as Critically Endangered (Ghogue 2010s). The taxon is known from only one locality. The extent of occurrence of *Z.microgyna* and the area of occupancy are estimated at about 4 km^2^ each. There is a dam built on the Sanaga River, the habitat of the species. Based on that threat, and the fact that the species is known only from one locality and the continuous decline of vegetation cover in the area, and extent and/or quality of habitat, *Z.microgyna* is here assessed as Critically Endangered. IUCN Red List Category: **Critically Endangered CRB1+2ab (ii, iii).**

## Discussion

The survey of rheophytic plants from Cameroon revealed 66 species distributed in 16 families, and in three major plant groups: 2 ferns, 8 monocotyledons, and 56 dicotyledons (Table 1). Among the monocotyledons only one grass and two sedges were recorded. Within the dicotyledons three shrub/small tree species, *Deinbollasaligna* Keay (Sapindaceae), *Ixoraeuosmia* K.Schaum (Rubiaceae) and *Pandanussatabiei* Huynh (Pandanaceae) were encountered (Table 1). According to van Steenis (1981), however, about half the species of rheophytes worldwide are trees and thus the paucity of trees in the current survey is surprising. Rheophytic woody plants (e.g., *Coffeacongensis* Froehn and *Breonadiasalicina* (Vahl) Hepper & Wood in the Rubiaceae) are known to occur outside the study area in Africa. Rheophyte diversity, according to some authors e.g., van Steenis (1981) and Hoyos-Gomez and Bernal (2018), is high in South East Asia and South America compared with tropical Africa. A survey of rheophytes of Africa by Ameka et al. (2002) found 53 rheophytes including 33 Podostemaceae species in Cameroon. This means that we have documented 10 more Podostemaceae and three other rheophytic species (from Cyperaceae: *Cyperusrheophyticus*, *C.tonkinensis*, and *C.cataractarum*) for Cameroon within the last 16 years. Further surveys are required across Africa for the full picture of rheophyte diversity and distribution to emerge.

**Table 1.** Number of rheophyte species and genera per family in Cameroon.

Family	Genus	Species
**Ferns**		
Lomariopsidaceae	1	2
**Dicotyledons**		
Acanthaceae	1	1
Amaranthaceae	1	1
Apocynaceae	1	1
Lamiaceae	1	1
Myrtaceae	1	1
Oxalidaceae	1	2
Podostemaceae	12	43
Rubiaceae	2	4
Sapindaceae	1	2
**Monocotyledons**		
Amaryllidaceae	1	1
Araceae	1	1
Cyperaceae	2	3
Melastomataceae	1	1
Pandanaceae	1	1
Poaceae	1	1

Invariably, the species encountered in the study have characteristic features that adapt them to their peculiar habitats, and enable them to persist in the harsh conditions of swift-flowing water, flush floods, torrents, and waterfalls. The leaves are lanceolate or narrow with a leaf index of at least 3 similar to what van Steenis (1978, 1981) observed while studying the rheophytes of the world. *Crinumnatans* and some other hydrophytes usually have ribbon-like leaves. The rheophytes encountered in Cameroon have firm but flexible stems which can withstand the tearing effect of swift-running rivers and streams. Their roots are strong or mat-rooted to hold them to their various substrates including rocks, gravel and boulders.

**Figure 26.**
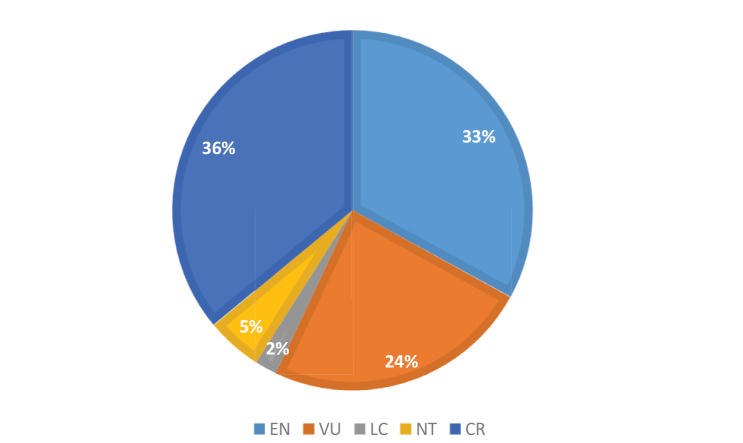
Percent distribution of rheophytes of Cameroon according to IUCN Red List Categories (CR-Critically Endangered; EN- Endangered; LC- Least Concern; NT- Near Threatened; and VU- Vulnerable).

The habitats of this unique biological group are, however, threatened by human activities. We show that about 36% of rheophytes are Critically Endangered (CR) in Cameroon and only 2% are considered to be of Least Concern (Fig. 26). Fifty three percent of Podostemaceae in Cameroon are in the CR group. There is the need to do more to protect the habitats of the rheophytes, particularly the Podostemaceae in Cameroon.

The habitats of rheophytes in Cameroon and indeed across Africa are threatened by the land use practices around the rivers, and the damming of the rivers for hydroelectric power (Cheek et al. 2015). One such land use practice, agriculture (arable farming), introduces agro-chemicals and silt to the rivers. This may make the river turbid, thus affecting photosynthetic ability and therefore the productivity of the plants (Ameka 2000). The agro-chemicals may also poison the plants. Timbering loosens the topsoil and during the wet season run-off water carries silt into the rivers; and the effect is the same as for silt from arable farming (Ameka pers. obs.) – turbid water which results in reduced productivity of submerged water plants. Mining is another land use practice affecting the plants and in particular alluvial mining in rivers may contribute silt into the rivers as suggested by Cheek et al. (2015) and Cheek and Lebbie (2018). The amount of silt from alluvial mining could be much higher than from agriculture because that from alluvial mining is generated *in-situ*, in the riverbed and, therefore, the effect may be more severe. Silt not only reduces photosynthesis efficiency but may also reduce or even prevent establishment of seedlings of rheophytes on rocks, particularly the members of Podostemaceae (Cheek et al. 2015). In some instances heavy metals such as mercury are used in the recovery of alluvial gold (Afum and Owusu 2016) and these may poison the plants. Indeed Philbrick and Crow (1983) and Philbrick and Novelo (1995) have provided evidence to show that there is a correlation between increased pollution (chemicals) and loss of Podostemaceae populations in South America.

The recent upsurge in dam construction effort in many African countries raises concern for the survival of rheophytes. In Cameroon, there are a number of dams built across rivers for hydropower, and efforts are continuing to build many more dams across a number of rivers: (http://www.theworldfolio.com/news/hydroelectric-projects/659/eroon); examples of these are:, Memve’élé hydroelectric dam on Ntem River(http://www.edennewspaper.net/memveele-hydroelectric-dam-is-60-complete-energy-minister/); Mekin hydro-electric dam on Dja River, Lom-Pangar hydroelectric dam on Lom River (https://www.internationalrivers.org/sites /default/files/attached-files/lp_factsheet.pdf); Menchum hydroelectric dam on Menchum River, and the Natchigal hydroelectric dam on Sanaga Rive1r at Edea; http://www.hydroworld. com/ articles/2013/11/cameroon-makes-deal-for-330-mw-nachtigal-falls-hydropower-project. html; and https://afrique.edf.com/en/edf-in-africa/news/a-new-phase-for-the-nachtigal-hydroelectric -project).

River rapids, cataracts, and waterfalls, are usually the preferred sites for dam construction for hydro-electric power, and also the habitats for many rheophytes, particularly the Podostemaceae. The rheophytes have become permanently submerged upstream of the dam due to flood water. The plants downstream are subjected to a different threat, that is, the change in flow rate and absence of flash floods below the dam (Cheek et al. 2015). In Ghana, two collecting localities of *Tristichatrifaria* (Podostemaceae) on the Volta River are now under lake water. Before the construction of the Akosombo dam on the Volta River in 1965, *T.trifaria* was collected at Kpando upstream of the dam. A second dam on the Volta River, down-stream of the Akosombo dam, was completed in 1982. *T.triticha* was collected on rocks in the river rapid just north of the Akuse dam (Ameka 2000). These two collecting sites have been lost because of the localities are permanently submerged (Ameka 2000). Thus dams threaten and /or endanger the very existence of rheophytic plants. We wish to draw attention to the threats posed by alluvial mining and dam construction to the survival of rheophytes and call on conservationists to do more to curb the indiscriminate damming of rivers, and alluvial mining across Africa. They must engage with policy makers in government and suggest alternative livelihoods for alluvial mine workers; and alternative green energy sources e.g., biofuels, biogas, and solar, instead of dam construction for hydropower.

## Supplementary Material

XML Treatment for Bolbitis fluviatilis

XML Treatment for Bolbitis heudelotii

XML Treatment for Lepidagathis alopecuroides

XML Treatment for Achyranthes talbotii

XML Treatment for Crinum natans

XML Treatment for Kanahia laniflora

XML Treatment for Anubias barteri

XML Treatment for Cyperus rheophyticus

XML Treatment for Cyperus tonkinensis C.B.Clarke var. baikiei

XML Treatment for Cyperus cataractarum

XML Treatment for Plectranthus cataractarum

XML Treatment for Calvoa stenophylla

XML Treatment for Eugenia dusenii

XML Treatment for Biophytum talbotii

XML Treatment for Biophytum zenkeri

XML Treatment for Pandanus satabiei

XML Treatment for Eragrostis barteri

XML Treatment for Ixora euosmia

XML Treatment for Ixora inundata

XML Treatment for Virectaria angustifolia

XML Treatment for Virectaria salicoides

XML Treatment for Deinbollia angustifolia

XML Treatment for Deinbollia saligna

XML Treatment for Dicraeanthus africanus

XML Treatment for Dicraeanthus zehnderi

XML Treatment for Djinga cheekii

XML Treatment for Djinga felicis

XML Treatment for Inversodicraea achoundongii

XML Treatment for Inversodicraea bosii

XML Treatment for Inversodicraea cristata

XML Treatment for Inversodicraea ebo

XML Treatment for Inversodicraea eladii

XML Treatment for Inversodicraea kamerunensis

XML Treatment for Inversodicraea ledermannii

XML Treatment for Inversodicraea ntemensis

XML Treatment for Inversodicraea tchoutoi

XML Treatment for Inversodicraea xanderi

XML Treatment for Ledermanniella aloides

XML Treatment for Ledermanniella batangensis

XML Treatment for Ledermanniella bifurcata

XML Treatment for Ledermanniella keayi

XML Treatment for Ledermanniella letouzeyi

XML Treatment for Ledermanniella linearifolia

XML Treatment for Ledermanniella monandra

XML Treatment for Ledermanniella musciformis

XML Treatment for Ledermanniella onanae

XML Treatment for Ledermanniella pollardiana

XML Treatment for Ledermanniella prasina

XML Treatment for Ledermanniella pusilla

XML Treatment for Ledermanniella sanagaensis

XML Treatment for Ledermanniella schlechteri

XML Treatment for Ledermanniella thalloidea

XML Treatment for Ledermanniella raynaliorum

XML Treatment for Ledermanniella variabilis

XML Treatment for Leiothylax quangensis

XML Treatment for Letestuella tisserantii

XML Treatment for Macropodiella heteromorpha

XML Treatment for Macropodiella pellucida

XML Treatment for Saxicolella flabellata

XML Treatment for Saxicolella laciniata

XML Treatment for Saxicolella marginalis

XML Treatment for Saxicolella nana

XML Treatment for Stonesia ghoguei

XML Treatment for Tristicha trifaria

XML Treatment for Winklerella dichotoma

XML Treatment for Zehnderia microgyna
